# Sleep and Circadian Interventions to Improve Athletes’ Mental Health, Mood and Well-Being: A Systematic Review and Meta-Analysis

**DOI:** 10.1007/s40279-025-02387-z

**Published:** 2026-01-27

**Authors:** Elie Walsh, Josh Leota, Minh Huynh, Sarah K. Liddle, Sean P. A. Drummond, Elise R. Facer-Childs

**Affiliations:** 1 Monash University, Clayton, VIC Australia; 2 La Trobe University, Melbourne, VIC Australia; 3 Division of Sleep and Circadian Disorders, Departments of Medicine and Neurology, Brigham and Women’s Hospital, Boston, MA USA; 4 Turner Institute for Brain and Mental Health, School of Psychological Sciences, Monash University, VIC, 270 Ferntree Gully Road, Notting Hill, 3168 Australia

## Abstract

**Background:**

Sleep and circadian interventions (e.g. interventions aimed at promoting circadian alignment and supporting stable well-entrained sleep–wake patterns) are predominantly implemented in athletes to improve performance, recovery and adaptation to travel. Emerging evidence from the broader population demonstrates that improving sleep and circadian health can also improve mental health, mood and well-being.

**Objectives:**

To evaluate the current evidence on the effectiveness of sleep and circadian interventions for improving mental health, mood and well-being in athlete populations.

**Methods:**

Seven databases (CENTRAL, CINAHL, Embase, MEDLINE, PsycINFO, Scopus, SPORTDiscus) were searched from inception to 23 September, 2024 in accordance with the Preferred Reporting Items for Systematic Reviews and Meta-Analyses (PRISMA) guidelines. Eligible studies involved athletes (participating in sport at any level) and evaluated sleep or circadian interventions aimed at improving sleep and/or circadian health, with mental health, mood or well-being outcomes. Sleep interventions directly manipulated sleep through behavioural, environmental or educational approaches. Studies without a comparator/baseline, using indirect methods (e.g. brainwave entrainment) to improve sleep or solely sleep restriction, were excluded. Two reviewers independently screened studies, extracted data, and assessed methodological quality and risk of bias using the Cochrane risk-of-bias tool and Joanna Briggs Institute checklist. Data were synthesised using a multi-level random-effects meta-analysis and pre-specified meta-regression.

**Results:**

A total of 21 sleep and circadian interventions have been implemented in sporting environments to improve the mental health of athletes. The interventions that have been investigated are constrained by limited sample sizes, a lack of female representation, a low-quality study design and inconsistent measurement of mental health, making it difficult to draw definitive conclusions of the efficacy of these strategies. The results of the meta-analysis showed that sleep and circadian interventions had a more substantial impact on positive affect compared to negative (*β* = 0.68, *p* < 0.001). The interventions were also found to be more effective in improving anxiety, tension and vigour compared to other subjective mood states. The limited interventions with a circadian component (e.g. light exposure) consistently found improvements in outcomes.

**Conclusions:**

Sleep and circadian interventions appear to have the strongest effects on positive affect, anxiety, tension and vigour in athletes. Future research should address the limitations of existing studies by focusing on diverse and representative samples, incorporating a longer term follow-up after interventions, using consistent mental health measurements and developing interventions specifically aimed at improving athletes’ circadian rhythms.

**Clinical Trial Registration:**

The protocol was registered in the PROSPERO database (#CRD42023467548).

**Supplementary Information:**

The online version contains supplementary material available at 10.1007/s40279-025-02387-z.

## Key Points

**Table:** 

This is the first systematic review to investigate the effects of sleep and circadian interventions on mental health, mood and well-being in athletes.
Sleep interventions are particularly effective at enhancing positive emotional states compared to negative affect.
Studies that integrated both sleep principles and behaviours supporting circadian health into their protocol showed positive results in improving mood and reducing anxiety.
Emerging evidence suggests appropriately timed light exposure may be an effective intervention for improving mood in athletes.
Future research should include diverse and representative samples, longitudinal designs, consistent mental health measurements and interventions aimed at strengthening circadian rhythms.

## Introduction

In 2019, the International Olympic Committee released a consensus statement emphasising the importance of investigating mental health in athletes and providing guidelines for assessing and managing sleep disorders and sleep concerns within this population [1]. Although competitive sports participation benefits mental health though camaraderie and physical activity, athletes also encounter sport-specific stressors that increase risk of mental ill-health [2]. Athletes experience mental ill-health at comparable rates to the general population [3, 4] with lifetime prevalence rates of mental health problems reaching up to 34% in elite athletes [3].

Mental health lacks a consistent definition in research. The World Health Organization describes mental health as “a state of well-being in which the individual realises his or her own abilities, can cope with the normal stresses of life, can work productively and fruitfully, and is able to make a contribution to his or her community” [5]. Various theoretical perspectives developed their own definition of mental health [6]. However, most perspectives agree mental health is not merely the absence of mental illness [7–10]. Although related, mental health is distinct from *mood*, defined as a prolonged core affect [11], and the more expansive *well-being*, defined as how well one functions across several domains [12]. Similarly, mental disorders are distinct from mental ill-health and are defined as a clinically significant disturbance in an individual’s cognition, emotion regulation or behaviour, reflecting dysfunction in underlying psychological, biological or developmental processes [13].

Athletes encounter sport-specific stressors that can cause sleep disturbances and circadian disruption, including late-night games, irregular training schedules, travel, competition stress, injury and illness [14–16]. Across studies of elite athletes, rates of sleep disturbance range from 50 to 78%, while 22–26% experience highly disturbed sleep [17, 18]. These findings are concerning as sleep is a fundamental component of athlete recovery and optimal performance [18, 19]. Sleep is also linked to athlete mental health; worse sleep quality, lower sleep duration and higher sleep disturbances are associated with increased depression, stress and anxiety symptomology [17–20].

An endogenous rhythm is an internal self-sustained cycle that regulates various physiological processes independently of external cues [21]. Humans have multiple endogenous rhythms, such as circadian rhythms that follow a 24-h cycle. Circadian rhythms synchronise our behaviour and physiology with the 24-h light–dark cycle, ensuring processes occur at optimal times [22]. The misalignment of endogenous circadian rhythms from external time cues is associated with poor athlete mental health, mood and well-being [20–25]. The circadian system can become disrupted by inconsistent light exposure. This disruption can desynchronise brain regions such as the amygdala and the lateral habenula, both of which are involved in mood regulation [26]. For example, circadian misalignment caused by rapid travel across time zones (i.e. jet lag [27]) can disrupt mood in athletes [28, 29].

Interventions addressing sleep and circadian disruption in athletes have been developed to improve performance, cognition, sleep and recovery [9, 30–34]. Sleep education, sleep hygiene, nutritional plans and daytime naps are among the most commonly used interventions for athletes [35, 36]. Systematic reviews suggest sleep plays an important role in some, but not all, aspects of athlete performance and recovery [30, 32–35]. Circadian interventions are common in clinical settings but are mostly limited to jet lag mitigation in athletes [37, 38]. The majority of interventions in sport have been focused on recovery and performance, with limited research having investigated how to improve mental health in athletes.

Sleep and circadian interventions provide an inexpensive and accessible option for sporting organisations to also improve the mental health of their athletes [14, 30, 35, 39]. A recent meta-analysis of sleep interventions reported small-to-moderate improvements in negative affect, including stress, irritability, psychological strain and fatigue, *g* = 0.54 (95% confidence interval [CI] 0.11, 0.97) [35]. However, the authors concluded that the effects were small and there was insufficient evidence regarding the efficacy of sleep interventions on mental health outcomes [35].

More recent meta-analyses in both clinical and general populations provide strong evidence that sleep interventions can significantly improve mental health. A meta-analysis of 49 randomised controlled trials (*n* = 5908) reported that non-pharmacological interventions produced moderate improvements in depression symptoms, with effects in clinical populations typically large and robust (standardised mean difference [SMD] = − 0.81, 95% CI − 1.13 to − 0.49, *p* < 0.001) compared with general populations (SMD = − 0.41, 95% CI − 0.51 to − 0.31, *p* < 0.001) [40]. Among university students, sleep interventions are associated with small but meaningful reductions in anxiety (SMD = − 0.23, 95% CI − 0.42 to − 0.03, *p* = 0.023) and depression (SMD = − 0.30, 95% CI − 0.51 to − 0.08,* p* = 0.008), although findings in this group are somewhat more variable [41]. Extending this evidence, a comprehensive review of randomised controlled trials demonstrated medium-sized improvements across multiple mental health outcomes (g = − 0.53, 95% CI − 0.68 to − 0.38, *p* < 0.001), including overall well-being, depression, anxiety and stress, though effects on stress appear less consistent across studies [42]. Importantly, none of these investigations focused on athletes, highlighting a critical gap in understanding whether similar interventions would be effective in populations facing unique challenges to sleep and circadian health.

This study aims to systematically review the literature on sleep and circadian interventions for mental health outcomes in athletes. Second, the review aimed to meta-analyse the effects of those interventions on various mental health outcomes. This review focuses on interventions aimed at manipulating sleep (e.g. sleep extension, sleep hygiene) or influencing circadian rhythms (e.g. melatonin ingestion, manipulating the timing and intensity of light exposure).

## Methods

This study followed the Preferred Reporting Items for Systematic Reviews and Meta-Analyses (PRISMA) guidelines [43, 44]. The protocol was registered in the PROSPERO database in September 2023 (#CRD42023467548).

### Data Source/Search Strategy

**Fig. 1:**
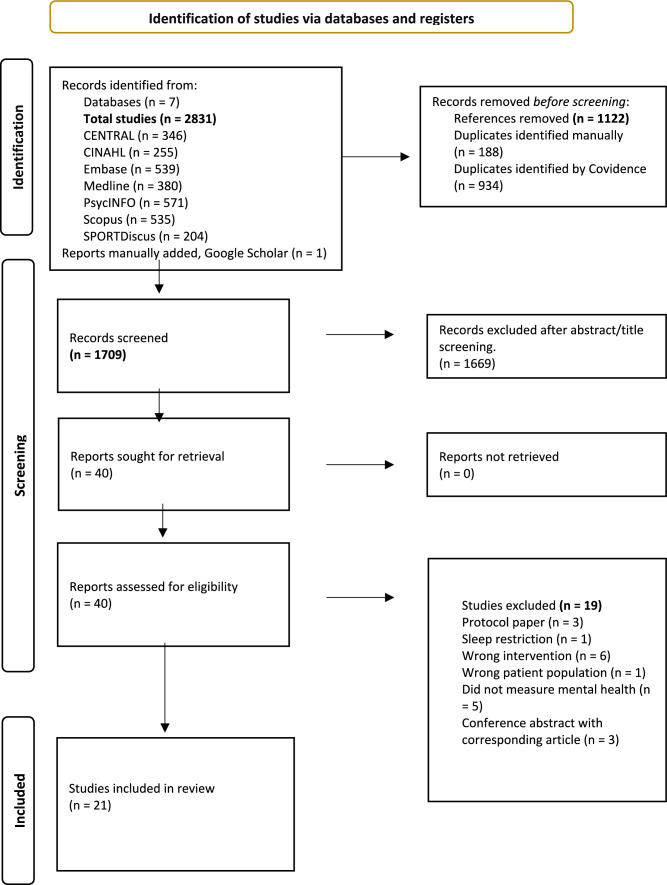
Preferred Reporting Items for Systematic Reviews and Meta-Analyses (PRISMA) flowchart for the selection of included studies

The search comprised sleep or circadian, athlete, intervention, study design and mental health terms. The search was implemented across seven databases (CENTRAL, CINAHL, Embase, MEDLINE, PsycINFO, Scopus, SPORTDiscus). The terms were searched from inception to 23 September, 2024 (the full search strategy, including search terms, is documented in the Table S1 of the Electronic Supplementary Material [ESM]). Search limits included non-human and non-English studies. Backward citation tracking was performed by screening the reference lists of relevant studies to identify eligible studies omitted in the original search. Figure 1 illustrates the search process.

### Eligibility Criteria

Eligible populations were individuals of any age who participated in sport (team or individual) at community, amateur, semi-professional or professional levels. Sleep interventions were defined as those in which sleep was directly manipulated or changed via behavioural, environmental or educational means (e.g. sleep extension, optimising the sleep environment, sleep hygiene education), consistent with prior definitions [31]. These were considered distinct from interventions with non-direct effects on sleep, such as brainwave entrainment, neurofeedback, physical activity, aromatherapy or infrared blankets. Circadian interventions were defined as interventions aimed at promoting circadian alignment and supporting stable well-entrained sleep–wake patterns in athletes.

Inclusion criteria were: (a) single- or multi-component interventions aimed at improving sleep and/or circadian health, including multi-component interventions that incorporated sleep restriction but were designed to improve overall sleep and well-being (e.g. Cognitive Behavioural Therapy for Insomnia (CBT-I), sleep regularity programmes) and (b) studies where a primary or secondary outcome assessed mental health, mood or well-being. Exclusion criteria were: (a) intervention studies without a comparator group or pre-intervention baseline period; (b) interventions aimed at improving sleep through indirect methods (e.g. brainwave entrainment); and (c) interventions involving sleep restriction as a sole component.

### Study Selection and Data Extraction

After removing duplicates, two authors (EW, JL) independently screened the titles and abstracts and then the full texts to determine whether the inclusion criteria had been satisfied. One study was identified for potential inclusion through reference searches of relevant studies. Disputes regarding inclusion were resolved by an independent third author (EF-C). One reviewer (EW) extracted data from eligible studies. Data included intervention design, time frame, follow-up, type of athlete, mental health outcome and measurement (Table S2 of the ESM). Conference abstracts meeting the inclusion criteria were retained for the narrative synthesis but were not included in the meta-analysis because of not undergoing a full peer review, incomplete reporting and insufficient methodological information.

### Quality Assessment

Two reviewers (EW, JL) independently evaluated the methodological quality and risk of bias of the full-text studies using the Cochrane risk-of-bias tool (ROB-2) for randomised trials, the ROB-2 for cross-over studies and the Joanna Briggs Institute checklist for pre-post and quasi-experimental studies (see Tables S3a-c of the ESM). The ROB-2 assesses five bias domains: randomisation process, deviations, missing outcome data, outcome measurement and reported results [45]. Each domain is rated as ‘low’ risk of bias ‘high’ risk of bias or ‘some concerns’, with the overall risk based on the least favourable domain, though this can be adjusted with justification. The ROB-2 for cross-over studies is adapted for cross-over designs. The Joanna Briggs Institute checklist evaluates the feasibility, appropriateness, meaningfulness and effectiveness of healthcare interventions and was used for non-randomised studies [43, 46].

### Meta-Analysis and Meta Regression

The meta-analysis included studies with both intervention and control groups, as well as single-group studies with pre- and post-intervention measurements. Standardised mean differences were calculated for two-group studies, and standardised mean changes were computed for single-group studies. The meta-analysis was conducted using the “metafor” [47] and “clubSandwich” [48] packages in R (R Core Team, 2021).

The included studies reported outcomes across several subgroups, often involving repeated measures on the same sample. To account for this hierarchical structure and within-subject correlation, we applied a multi-level random-effects meta-analysis. The dependency of effect sizes within studies was addressed by replacing the variance with the variance–covariance matrix of the estimates for outcomes reported from the same study. A block-diagonal covariance matrix was estimated for each study, assuming a correlation of *r* = 0.50 between outcomes. Outcomes of multiple studies were combined when they measured conceptually similar constructs. Outcomes that were reported by two or fewer studies could not be combined because of an insufficient conceptual overlap and were excluded from the analysis (confidence [*n* = 2], motivation [*n* = 2], negative affect [*n* = 2] and positive affect [*n* = 1]).

We combined effect sizes from single- and two-group studies using a random-effects model to account for heterogeneity across studies. I^2^ and tau^2^ statistics quantified the degree of heterogeneity. Subgroup analyses explored differences between study designs (single-group vs two-group). Pooled effect sizes were calculated as a weighted average of individual effect sizes, with weights determined by the inverse of the sum of the within-study and between-study variance.

We also conducted a single pre-specified meta-regression with affect valence (positive vs negative) as the moderator to probe whether effects differed by outcome valence. We combined outcomes by affect valence, a core organising dimension of emotion [49]. We operationalised affect valence as how good or bad an affective experience feels [50]. Consistent with classic evidence, worry and anxiety correlate with negative affect but not with positive affect [51]. Accordingly, combined outcomes indexing unpleasant states (e.g. anger, anxiety, confusion, depression, stress, tension, total mood disturbance, worry, negative mood/affect) were mapped to negative affect, and combined outcomes indexing pleasant states (e.g. confidence, motivation, positive affect, vigour/energy) to positive affect. We report the moderator coefficient (β), 95% CI, *p*-value and fitted values with 95% prediction intervals (PIs).

## Results

The database search yielded 2831 records. After removing duplicates and initial screening, 40 full texts were examined. Nineteen full texts were excluded for not meeting the inclusion criteria, leaving 21 studies for review (Fig. 1).

### Description of Included Studies

Included studies comprised conference abstracts (*n* = 8) and full-text articles (*n* = 13). All 21 studies were included in the narrative synthesis, whereas only the full-text articles (*n* = 13) were eligible for inclusion in the meta-analysis. Studies were reviewed based on study design, intervention type, intervention characteristics, location, sport type, participants, outcome, measurements and key results. Sample populations consisted of athletes from amateur to professional sporting levels from various countries. Thirteen studies were conducted in the USA, Canada or Australia, one study was conducted in Germany, one in Japan and six studies did not report the study location. Athletes also participated in various team (*n* = 11) and individual sports (*n* = 6), and four studies did not specify the sport type. A total of 558 athletes were included (mean age = 21.36 years; 22.7% women). The number of participants in each study ranged from 7 to 85. Nine studies investigated exclusively male athletes, and one study investigated exclusively female athletes, while seven studies included mixed-sex samples and four did not report sex distribution. Ten intervention studies used a mixed-component methodology. The most common methodologies were sleep extension, sleep hygiene, sleep education and light regulation. Study designs included within-subject pre-post designs (*n* = 8), randomised cross-over designs (*n* = 6), non-randomised cross-over or counterbalanced cross-over designs (*n* = 3) and parallel-group randomised controlled trials (*n* = 4). The intervention period varied between 1 and 70 days (mean = 15.36 days, median = 5 days), with the intervention follow-up being ≤ 1 week in 12 studies.

All studies measured the impact of sleep or circadian interventions on mental health, mood and/or well-being outcomes. Mood was the most commonly assessed construct, with 13/21 (61.9%) studies including a mood measure (11/21 used the Profile of Mood States [POMS], 1/21 used the Brunel Mood Scale [BRUMS], and 1/21 used a Wellness and Mood States questionnaire). Other less commonly investigated measurements included overall mental health (*n* = 1), stress (*n* = 2), well-being (*n* = 2), depression symptoms (*n* = 1), anxiety symptoms (*n* = 3), motivation (*n* = 1), irritation (*n* = 1), and positive and negative affect (*n* = 1; see Table S4 of the ESM). Several studies assessed more than one of these constructs, so the counts across outcome categories sum to more than 21. No studies used athlete-specific measurements of mental health or ill-health or clinical interviews of mental ill-health symptomology.

### Results of Types of Interventions

#### Sleep Extension Interventions

Twelve studies employed an intervention that increased sleep opportunity, with the aim to increase total sleep time (TST) throughout the night (*n* = 5) and/or through napping (*n* = 7) [52–56]. The only all-female study [57] examined track athletes. The intervention increased time in bed by 1 h per night for 1 week [57]. Minimal information concerning the intervention was included, such as whether sleep extension was individualised or if napping was included. However, the results showed that TST increased and total mood disturbance decreased compared with baseline.

Three studies employed an intervention that increased sleep opportunity to 10 h per night [52, 53, 58]. Mah et al. [52] implemented a 5- to 7-week sleep extension intervention in 11 professional college basketball players. The study was undertaken in real-world conditions, with the participants continuing to participate in their everyday routines. Following the intervention, participants reported decreased total mood disturbances (10.36 ± 9.62) compared with baseline (13.76 ± 17.17). Mah et al. [58] also conducted a 5-day randomised controlled trial with a similar protocol in professional baseball players. Participants in the sleep extension group reported a 33.8% decrease in tension on the POMS compared with the control group, but there was no information for other POMS sub-scores. Ritland et al. [53] employed a 10-h sleep opportunity randomised controlled trial in military tactical athletes during a training camp. Outcomes included motivation and anxiety symptoms prior to motor and cognitive performance tasks. The intervention group showed a significant increase in actigraphic-derived TST from baseline (mean* ∆* = 1.36 ± 0.71 h), whereas the control group did not show a significant increase in TST from baseline (mean ∆ = − 0.25 ± 0.78). There was no difference in anxiety, but the intervention group reported higher motivation for the cognitive task (mean = 4.44 ± 11.85) compared with the control group (mean = 7.04 ± 13.63, *p* = 0.003).

Roberts et al. [54] used an individualised approach to increase sleep opportunity. In a counterbalanced cross-over design, triathletes and cyclists undertook three nights of (a) time in bed extension by 30%, (b) time in bed restriction by 30% or (c) habitual sleep. Participants had a TST of between 8 and 9 h on the three nights in the extension condition nights (mean = 8.37 ± 0.76 h), significantly higher than the habitual sleep and restricted sleep conditions (*p* < 0.001). Only the vigour POMS sub-score was higher on day 4 of the intervention between the sleep extension and habitual sleep conditions (*p* < 0.025). Taken together, the current evidence suggests that extending the sleep opportunity during the night could improve aspects of an athlete’s mood and motivation.

##### Sleep Extension Interventions: Napping

Three studies investigated the effects of a nap opportunity intervention on acute athlete mood [55, 56, 59]. Souabni et al. [56] evaluated the effectiveness of a 40-min nap opportunity in professional basketball athletes who were not habitual nappers using a cross-over design. There was a significant reduction in anxiety (*p* < 0.01, *d* = − 1.26) and a significant increase in vigour in the napping condition compared with the control condition (*p* = 0.03, *d* = 0.8).

Boukhris et al. [59] investigated whether a 90-min nap was more effective than a 40-min nap for improving mood. Amateur team sport athletes were allocated to a no nap, 40-min nap or 90-min nap condition with a 72-h washout period between conditions. The POMS was used to investigate mood pre- and post-nap. Both nap conditions significantly improved athlete mood compared with the control condition. A 90-min nap was more effective in lowering tension (6.8 ± 3.5 vs 7.4 ± 2.7), depression (6.4 ± 7.2 vs 7.9 ± 8.0) and total mood disturbance (10.9 ± 18.1 vs 18.0 ± 19.6) compared with a 40-min nap (*p* < 0.05).

Last, Bentouati et al. [55] investigated the combined effects of napping and listening to motivational music on mood in karate athletes. Participants were randomly assigned to one of three conditions: (a) 30-min nap opportunity; (b) a warm-up with self-selected motivational music; or (c) a combination of (a) and (b), with a 7-day washout period between conditions. All conditions improved mood (*p* < 0.05).

The current evidence suggests napping improves acute mood in athletes. Further evidence is needed to determine whether napping improves long-term mood in athletes and whether the timing of naps moderates these effects.

##### Sleep Extension Interventions: Multicomponent (Regularity)

Only one study investigated a sleep regularity intervention (i.e. regular sleep–wake schedule) in athletes [60]. Forty-six athletes were randomised to a sleep consistency or sleep extension intervention for 2 weeks, with a 2-week washout period between conditions. There was no change in mood on the POMS for either intervention compared to baseline. There was no information provided on whether sleep duration increased, or whether sleep regularity improved during these interventions. More evidence is needed to determine whether increasing sleep consistency improves mood.

#### Sleep Hygiene Interventions

Nine studies investigated the effects of variations of a sleep hygiene intervention on sleep and subsequent mental health outcomes in athletes. The interventions endorsed similar recommendations, which included restricting light before bed, avoiding devices and optimising the bedroom environment for sleep. Three studies included sleep education as part of the intervention protocol [61–63], two studies implemented a brief sleep hygiene intervention [64, 65] and three studies included a light regulatory component through the use of blue light blocking glasses and/or light therapy devices [66–68]. The interventions are detailed below.

Two studies investigated acute sleep hygiene interventions on mood in tennis and soccer players [64, 65]. One study implemented the protocol (no light stimulation was allowed ~ 15–30 min prior to bedtime, optimise sleep environment, sleep window) following a late-night soccer match [64]. There was no difference in next-morning overall stress between conditions. The Duffield et al. [65] protocol included sleep hygiene strategies and assisted sleep tools such as cold-water immersion and full body compression. This study found no difference on the POMS vigour sub-score between the intervention and control conditions. The current findings indicate that acute sleep hygiene strategies alone may not provide improvements in athlete stress and vigour.

#### Sleep Hygiene and Sleep Education

Interventions that investigated sleep hygiene over a longer period showed mixed results. Van Ryswyk et al. implemented a 6-week sleep optimisation period in male Australian Rules Football athletes [62]. The intervention included a 1-h group education session presented by an expert sleep physician, feedback to improve sleep, and a 1-h mid-programme education session. Sleep was measured using actigraphy devices and self-report sleep diaries, and participants completed pre- and post-intervention mood questionnaires including the POMS and the Perceived Stress Scale [69]. Only vigour improved following the intervention (*p* < 0.001) increasing from 11.6 ± 6.4 to 15.9 ± 4.9 post-intervention. Participants self-reported higher sleep duration and sleep efficiency; however, there were no significant changes in objective sleep outcomes. Bonnar et al. [63] also investigated the effects of a group education workshop, daily feedback, and a 30-min one-on-one session with a clinical psychologist in a sample of e-sport athletes over 2 weeks. The intervention did not significantly improve depression (27.33 ± 1.85 to 27.40 ± 1.99) or anxiety (44.96 ± 3.30 to 43.78 ± 3.39).

Lever et al. investigated the effect of a one-night sleep education intervention combined with sleep hygiene recommendations and mindfulness on mood, worry, anxiety and self-confidence in adolescent tennis players prior to a tournament [61]. No statistically significant between-group differences were detected within 5 days of the intervention. However, in the control group, worry scores decreased from 8.61 ± 1.00 to 7.92 ± 1.11 and confidence from 28.18 ± 5.87 to 24.37 ± 5.44 (both *p* < 0.05), indicating greater pre-competition worry and lower confidence than at baseline. These findings indicate that sleep education and hygiene recommendations may not be effective in improving mood, anxiety, depressive symptoms and stress, but combined with mindfulness, could be a protective strategy for worry related to competition.

#### Light Regulation

Fowler et al. investigated whether sleep hygiene and exposure to and avoidance of light reduced the effects of jet lag and eastward international air travel in athletes [68]. Participants were instructed to seek natural outdoor light or utilise wrap-around sunglasses to minimise exposure to light at specific times. If natural light was not available, artificial bright light emitting glasses were used (506 lx). The intervention mitigated the adverse effects of travel, including mood symptoms. The control condition reported increased total mood disturbance (4.2 ± 8.2 to 8.5 ± 9.2) and decreased motivation (2.3 ± 0.8 to 1.4 ± 0.8) on the third day after travelling compared with baseline, and the intervention group reported no change.

Two studies presented as conference abstracts [66, 67] investigated interventions using sleep optimisation techniques such as daily napping, increasing sleep, electronic device restriction and wearing blue light-blocking glasses. Using the POMS to measure mood, Charest et al. found the intervention increased vigour and reduced total mood symptoms in speed skaters (*p* ≤ 0.05) [67] and Bender et al. found their intervention reduced depressive symptoms in Canadian National Team rowers and increased vigour and reduced total mood symptoms in Canadian National Team curlers (*p* < 0.05) [66]. These findings highlight the potential for including light regulation components (e.g. blue light blocking glasses, bright light glasses or boxes, and regulating natural light exposure) in sleep interventions to improve mood in athletes. However, as sample sizes were small and the studies reported were not available in full-text articles, it is difficult to determine whether other factors could have contributed to these results.

#### Behaviours Supporting Circadian Health

One study included an intervention that supported circadian health promoting a more “morning-type” lifestyle for university soccer players [70]. The intervention included a protein-rich breakfast, morning sunlight exposure and low-colour-temperature lights (e.g. incandescent lights). Participants self-reported a greater morningness preference following the intervention compared with baseline, and lower irritation scores at the 3-month follow-up (*z* = − 2.74, *p* = 0.006). However, it is important to consider the lack of an objective circadian measurement (e.g. melatonin assessment) when interpreting these results [71].

#### Device Restriction

One study investigated a single-component intervention of whether electronic device restriction after 10.00 p.m. improved mood in a sample of adolescent athletes [72]. No differences in mood were reported. Device restriction as a sole intervention may not be effective in improving mood in athletes.

### Results of Meta-Analysis

A meta-analysis on the full-text studies (*n* = 13) was conducted to examine the effects of sleep and circadian interventions on various mental health outcomes, including mood and well-being. Data from multiple studies were aggregated when they assessed conceptually similar constructs. The results of the multi-level random-effects meta-analysis are provided in Table 1. The imprecision of the estimates was expressed with 95% compatibility CIs, calculated from a *t*-distribution with denominator degrees of freedom given by the inner level of the random-effects structure. Table 1 also includes 95% PIs, which convey the likely range of the true outcome values in similar future studies, as well as standard deviations and 95% CIs (calculated using the Q-profile method) to quantify the between-study and between-group heterogeneity for each pooled estimate.

**Table 1:** Pooled effect size for each outcome following the meta-analysis of the effects of sleep and circadian interventions on mood and mental health outcomes in athletes

Outcome	Number of		Pooled effect			Variation between			
	Studies	Samples	Beta	95% CI	95% PI	Studies	Samples	*τ*^2^	*I*^2^
Anger	4	5	− 0.34	− 0.85, 0.16	− 1.29, 0.6	0.07 (0, 2.84)	0.1 (0, 1.65)	0.29	0.95
Anxiety	4	5	− 0.78	− 1.56, 0	− 2.51, 0.94	0 (0, 2.87)	0.62 (0.08, 4.78)	0.72	0.92
Confusion	4	5	− 0.22	− 0.51, 0.07	− 0.51, 0.07	0 (0, 0.41)	0 (0, 0.2)	0.02	0.77
Depression	5	6	− 0.08	− 0.52, 0.35	− 0.97, 0.8	0.15 (0, 1.99)	0 (0, 0.87)	0.32	0.95
Mood	7	8	− 0.36	− 0.93, 0.21	− 1.79, 1.07	0.41 (0, 2.6)	0.04 (0, 1.7)	0.64	0.95
Tension	3	4	− 0.71	− 1.08,− 0.34	− 1.16,− 0.27	0.02 (0, 1.68)	0 (0, 0.48)	0.05	0.88
Vigour	5	6	0.72	0.41, 1.02	0.41, 1.02	0 (0, 0.75)	0 (0, 0.33)	0.11	0.75
Worry and stress	2	2	− 0.16	− 0.55, 0.22	− 0.55, 0.22	0 (0, 9.27)	0 (0, 9.27)	0.06	0.85

*CI* confidence interval, *PI* prediction interval

As shown in Table 1, anger showed high dispersion (*τ*^2^ = 0.29; *I*^2^ = 0.95) with a wide PI spanning benefit and harm (− 1.29 to 0.60), so it was interpreted cautiously and context dependently. Anxiety similarly displayed substantial heterogeneity (*τ*^2^ = 0.72; *I*^2^ = 0.92) with a very wide PI (− 2.51 to 0.94). Confusion had low absolute heterogeneity (*τ*^2^ = 0.02) and a PI that mirrored the CI (− 0.51 to 0.07), though *I*^2^ was moderate to high (0.77), indicating the between-study spread was small on the standardised scale. Depression showed high dispersion (*τ*^2^ = 0.32; *I*^2^ = 0.95) with a broad PI (− 0.97 to 0.80). Overall mood (total mood disturbance) exhibited substantial heterogeneity (*τ*^2^ = 0.64; *I*^2^ = 0.95) and a very wide PI (− 1.79 to 1.07), so PIs were emphasised to avoid over-generalisation. Tension showed modest dispersion (*τ*^2^ = 0.05; *I*^2^ = 0.88) and a PI that did not cross zero (− 1.16 to − 0.27), supporting a consistently beneficial reduction. Vigour showed relatively lower dispersion (*τ*^2^ = 0.11; *I*^2^ = 0.75) with a PI that mirrored the CI (0.41 to 1.02), indicating reasonably consistent gains. Worry/stress showed modest dispersion (*τ*^2^ = 0.06; *I*^2^ = 0.85) with a PI paralleling the CI (− 0.55 to 0.22), but with *k* = 2 these estimates are imprecise and should be treated as tentative.

**Fig. 2:**
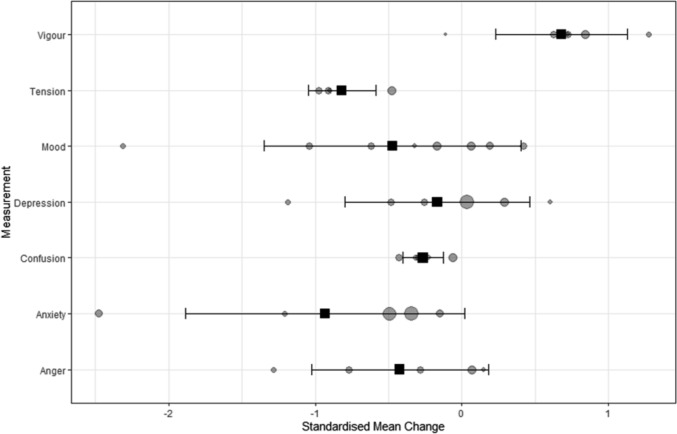
Standardised mean change in mood and mental health outcomes in athletes following sleep and circadian interventions: pooled and individual study effect sizes. Squares denote pooled average effect sizes (*β*) with 95% confidence intervals; circles denote individual study effect sizes, with the marker size proportional to the sample size. The plot shows the average effect size for each subgroup alongside individual study estimates

Forest plots are shown in S5 of the ESM. The plots illustrate the effect of sleep and circadian interventions on the various mental health outcomes (S5.1–S5.8 of the ESM). The meta-analysis results are visually represented in Fig. 2, which plots the average effect sizes for each subgroup alongside individual study effect sizes. The beta values, shown as squares, represent the pooled average effect sizes, while individual study effect sizes are indicated by circles, with the size of each circle reflecting the corresponding sample size (larger circles indicate larger samples). The ‘Worry and Stress’ subgroup was excluded from the analysis because of insufficient data (only two studies), ensuring the results focused on subgroups with more robust data.

Using the positive and negative affect groups in Table 2, a meta-regression was performed to determine whether this moderated the effect score. Positive affect was, on average, 0.857 points higher than negative affect, and this was statistically significant, *p* < 0.001 (see Table 3).

**Table 2:** Mood and mental health outcomes from the meta-analysis, categorised into negative affect and positive affect following sleep and circadian interventions in athletes

Negative affect		Positive affect	
Metric	*n*	Metric	*n*
Anger	7	Confidence	2
Anxiety	5	Motivation	2
Cognitive anxiety	2	Positive affect	2
Confusion	7	Vigour	9
Depression	8		
Mood	4		
Negative affect	2		
Somatic anxiety	2		
Stress	1		
Tension	5		
Total mood disturbance	8		
Worry	2

**Table 3:** Meta-regression examining the moderating effect of affect valence (positive vs negative) on the impact of sleep and circadian interventions on mental health outcomes in athletes

Term	Beta estimate	SE	*P* value
Intercept	− 0.345	0.112	0.002
Affect [positive–negative]	0.857	0.185	< 0.001

*SE* standard error

### Risk of Bias/Quality Assessment

The complete summary of the risk of bias for each study is presented in Table S3 of the ESM. Only full-text articles were reviewed for quality assessment (*n* = 13).

Overall, we reported a “high” risk of bias in the analysis of randomised cross-over trials, resulting from missing outcome data, selection of reported results, and deviations from the intended protocol. We also noted an overall bias of “some concerns” for the randomised controlled trials. Because of the nature of the interventions or the chosen designs, the blinding of personnel and participants to conditions was often not conducted. Attrition was also not typically reported. Most studies did not publish a protocol on trial registries, making it difficult to assess reporting bias. These methodological omissions and high risk of bias led to the downgrading of the evidence quality for most of the included intervention types.

No study was excluded from the analysis based on the Joanna Briggs Institute critical appraisal checklist for the single group pre-post design studies [46]. The majority of domains included a majority of “yes” listed, but domains such as the inclusion of control groups and multiple outcome measurements reduced evidence quality in these studies.

## Discussion

### Current State of the Literature

This is the first review to systematically assess and summarise the current sleep and circadian intervention studies aimed to improve mental health, mood or well-being in athletes. This review found sleep plays a pivotal role in improving subjective mood states [73–77]. The meta-analysis demonstrated that sleep and circadian interventions were associated with moderate reductions in anxiety (*β* = − 0.78, 95% CI − 1.56 to 0.00) and tension (*β* = − 0.71, 95% CI − 1.08 to − 0.34), and moderate increases in vigour (*β* = 0.72, 95% CI 0.41–1.02), with consistent effects across studies and samples. These results highlight the effectiveness of sleep and circadian interventions in alleviating stress-related symptoms and promoting positive emotional states. However, outcomes such as anger, depression, mood and worry/stress showed mixed or non-significant effects, with wide CIs and PIs reflecting variability and imprecision in the findings. These inconsistencies may stem from differences in study design, participant characteristics or intervention protocols across the included studies. Most studies reviewed here were multi-compositional with varied study designs; therefore, it is difficult to identify causality between sleep and circadian interventions and athletes’ mood.

Previous meta-analyses in non-athlete populations have generally reported moderate and consistent benefits of sleep interventions for depression, anxiety and stress, with some showing larger effects in clinical populations. In contrast, our findings indicate smaller and more variable effects in athletes [40–42]. This discrepancy may partly reflect differences in sample characteristics. Prior reviews often included a large number of clinical populations, where improvements tend to be greater, particularly among individuals with existing sleep disorders such as insomnia [40, 42]. The findings of this review are also somewhat comparable to research examining psychological sleep interventions in university students, which has similarly reported smaller but positive effects on reducing symptoms of anxiety and depression [41]. Notably, unlike much of the existing work, this review highlights clear and consistent benefits for positive mood dimensions such as vigour and positive affect, which have been less commonly examined in non-athlete populations.

### Effects of the Interventions

A key challenge in this review was determining the most effective interventions for improving outcomes, as many studies used multi-component approaches that combined elements such as sleep hygiene, education and napping, making it difficult to isolate the impact of individual components. However, interventions including a circadian component (i.e. manipulation of light) reported the most consistent improvements, including reduced irritation [74], depressive symptoms [67], and total mood symptoms and increased vigour [67]. Though the number of studies were limited, there is promising evidence that manipulating light exposure through blue light-blocking glasses or recommending behaviours that promote circadian health can improve mood in athletes. This finding is consistent with previous studies examining the effect of light on mood in clinical populations [78, 79]. Light has been found to directly and indirectly (via the circadian system) affect the mood circuits in the brain, with the timing, frequency, duration and intensity of light determining whether these effects are positive or negative [79]. Light therapy may also reduce depressive symptoms in non-seasonal depression [80].

### Knowledge Gaps

#### Adherence

Few studies reported adherence to intervention protocols. Nonetheless, the few studies reporting low adherence rates reported no improvements in mood outcomes following the intervention. For example, in one study examining the effect of device restriction on mood, only 7 out of 44 (15.9%) participants adhered entirely to the intervention protocol [72]. Adherence reporting is essential as it can assist in disentangling conflicting results. Future intervention research should prioritise the reporting of adherence rates.

#### Mental Health Measurement

Consistent with previous findings of systematic reviews on mental health, this study highlights the difficulties in conceptualising, operationalising and defining mental health in the intervention studies [81]. Overall, mood was the most investigated outcome measured predominantly by the POMS. Measuring mood states is a well-established mental health indicator [82]. However, by conceptualising mental health as solely a function of mood, we ignore other constructs that contribute to one’s mental health. In clinical practice, determining a patient’s mental health status often involves a periodic mood assessment as it is well recognised that mood is variable and fluctuates based on both internal and external factors [83, 84]. Moreover, with the exception of the vigour and fatigue items, the POMS is not particularly sensitive to the acute effects of sleep loss [85, 86]. Therefore, it is unsurprising only the vigour scale showed consistent improvements with sleep extension in the studies reviewed. Future research needs to more clearly and specifically conceptualise and measure mood. When conducting intervention studies, researchers should consider moving beyond traditional pre-post mood scales and incorporating an ecological momentary assessment methods, whereby brief mood ratings are collected repeatedly in athletes’ everyday environments (e.g. via smartphone prompts). An ecological momentary assessment allows a real-time context-specific assessment and explicitly captures the variability and reactivity of mood over time, providing a more sensitive and ecologically valid index of mental health than isolated mood snapshots [87].

The average participant age was 21.36 years, with many athletes recruited in early adulthood or late adolescence. Adolescence is a time of variable mood states and a critical period for emotional and physical development [84, 88], which could confound some of the reported findings. Similarly, the POMS was developed for detecting transient mood states. The length of the POMS also restricts regular monitoring in the sports environment, which is essential when capturing mental states before and after performance that may be associated with mental ill-health and psychiatric disorders [8]. Mental health is a multi-dimensional and complex construct that captures numerous elements beyond just transient changes in mood. Other factors such as balance, identity, agency and social connectedness were not captured in the present studies [89]. Future research should develop new measurements of athlete mental health that can address these limitations and assess more than just mood and the absence of mental illness.

#### Lack of Representation of the Female Athlete Population

There was a lack of female athlete representation in the studies reviewed. Sleep and circadian interventions may vary in efficacy across sexes [35]. One study found that elite female football players are at a greater risk of poor mental health when experiencing low sleep efficiency, sleep irregularity or circadian misalignment relative to their male counterparts [90]. These findings support the need to further assess sleep and circadian interventions in female athletes [90].

#### More Research to be Done Targeting Circadian Rhythms

Circadian rhythms, as a therapeutic target for improving mental health [23], are an untapped resource in the sporting population. Circadian interventions are occasionally used to mitigate jet lag and enhance performance, but research on their impact on athlete mental health is limited [91]. Although studies that utilised a circadian intervention found improvements to subjective mood states of athletes, they did not include objective measurements of circadian rhythms [71]. Previous literature has shown relationships between worse psychological well-being and an eveningness chronotype in athletes [92, 93] with the morningness preference typically associated with better well-being, more social support and mindfulness [94]. Therefore, circadian interventions aimed at advancing the circadian phase or promoting a preference towards morningness may have beneficial effects on athlete mental health, mood and well-being.

### Implications

These findings have important applied implications for coaches, sport psychologists and athlete support staff in elite sport. Effective implementation of sleep and circadian interventions should involve a multi-disciplinary team, including sleep clinicians, researchers, physicians, sport scientists, dietitians, psychologists and physiotherapists, to ensure that sleep data are accurately monitored, education is evidence based, and strategies are tailored to athlete needs [95]. Interventions should be carefully timed to align with training schedules, competition demands and travel-related circadian disruptions to maximise adherence and feasibility. Beyond reducing negative mental health symptoms, our findings highlight the potential of these interventions to enhance positive mood dimensions, such as vigour and positive affect, which are particularly relevant for athlete well-being and performance. Therefore, future research should incorporate broader measures of positive mental health, such as the Mental Health Continuum Scale [96] or measures of confidence and motivation, to better capture the full impact of these interventions on athlete well-being. Additionally, implementation should consider sex differences, chronotype preferences and insomnia status, as these factors may influence intervention effectiveness.

### Strengths and Limitations

This review provides a comprehensive and up-to-date search of interventions examining the effect of improving sleep and circadian rhythms on various subsequent mental health outcomes in athletes. This is the first systematic review examining the effect of circadian interventions on mental health in athletes. However, several limitations should be considered. First, the review is limited by the multi-component nature of the interventions as well as variability in mental health measurements. Second, the review was limited to studies published in English. Third, the review included conference abstracts that lacked sufficient methodological details, making it difficult to assess bias and study reliability. However, including conference abstracts broadened the review by capturing studies from journals, conference proceedings and grey literature. This ensured a representative evidence sample and highlighted unpublished studies, revealing novel intervention types and key results. The authors of these conference abstracts should aim to publish the full written studies in peer-reviewed journals to strengthen the evidence base in this area. Future research should also examine potential differences in intervention effects across specific athletic populations (e.g. professional vs amateur athletes) once sufficient studies are available.

Given the limited number of eligible studies for several outcomes, single-group pre-post and two-group controlled trials were pooled to maximise statistical power and stability. While this approach is common when synthesising a diverse evidence base, it may obscure potential differences between study designs. As the literature grows, future meta-analyses should examine single-group and controlled trials separately to better understand design-specific effects.

## Conclusions

Overall, research on sleep and circadian interventions targeting mental health outcomes in athletes remains limited. The existing literature primarily focuses on mood outcomes, with few studies analysing broader mental health. Furthermore, the research lacks female representativeness, and the mixed results restrict the generalisability and applicability of findings. Despite these limitations, the evidence suggests that sleep and circadian interventions that combine foundational sleep principles with behaviours supporting circadian health, particularly sleep extension and light exposure, hold significant promise for improving athlete mood and reducing anxiety. Sleep and circadian interventions also appear more effective in enhancing positive affect than reducing negative affect, with notable benefits for reducing anxiety and tension and increasing vigour compared to other subjective mood states. Future research should explore circadian rhythms, sleep regularity and light interventions, leveraging wearable technology to assess long-term outcomes. High-quality intervention studies with extended follow-up periods and greater representation of female athletes are essential.

## Supplementary Information

Below is the link to the electronic supplementary material.

Supplementary file1 (DOCX 30 KB)

Supplementary file2 (DOCX 3475 KB)

Supplementary file3 (DOCX 54 KB)

## Declarations

### Availability of data and material

All data relevant to the study are included in the article or uploaded as supplementary information.
