# Supplementary material for: Sleep and Circadian Interventions to Improve Athletes’ Mental Health, Mood and Well-Being: A Systematic Review and Meta-Analysis
Source: Sports Med. 2026 Jan 27;56(4):997–1011. doi: 10.1007/s40279-025-02387-z (PMC13124852; doi:10.1007/s40279-025-02387-z)

**Electronic Supplementary Material**

| **Table S1a, PICO search strategy for all databases** | | | |
| --- | --- | --- | --- |
| **Population** | **Intervention** | **Comparator** | **Outcome** |
| athlete OR sports OR olympian OR players | sleep OR nap OR insomnia OR rest activity OR insomnia OR circadian OR "diurnal rhythm" OR "body clock" OR "biological rhythm" OR "biological clock" OR chronobiology OR chronotype OR "light exposure" OR zeitgeber OR melatonin OR "core body temperature" OR sunlight | intervention OR program OR trial OR baseline OR workshop OR randomize | "mental health" OR depression OR anxiety OR wellbeing OR mood OR "mental ill-health" OR psychological OR stress OR "mental state" |

**Table S1b, Record hits for databases searched. The literature search examined databases from their inception until 23^d^ September 2024.**

| **Database** | **Records** |
| --- | --- |
| Medline | 380 |
| Embase | 539 |
| PsycINFO | 71 |
| Scopus | 535 |
| CINAHL | 255 |
| SPORTDiscus | 204 |
| CENTRAL | 346 |

**Table S1c, Complete searches**

***Central (Cochrane)***

Records: 346 (*with limits)*

ID Search

#1 MeSH descriptor: [Sleep] this term only

#2 MeSH descriptor: [Mental Health] this term only

#3 MeSH descriptor: [Circadian Clocks] this term only

#4 MeSH descriptor: [Athletes] explode all trees

#5 (sleep or nap or insomnia or (rest NEXT activity) or insomnia or circadian or "diurnal rhythm" or "body clock" or "biological rhythm" or "biological clock" or chronobiology or chronotype or "light exposure" or zeitgeber or melatonin or "core body temperature" or "sunlight"):ti,ab,kw

#6 ("mental health" or depressi* or anxi* or wellbeing or mood or "mental ill-health" or psycholog* or stress or "mental state"):ti,ab,kw

#7 (athlete* or sports* or Olympian or players):ti,ab,kw

#8 (interven* or program or trial or baseline or workshop or randomiz*):ti,ab,kw

#9 #1 OR #3 OR #5

#10 #2 OR #6

#11 #4 OR #7

#12 #9 AND #10 AND #11 AND #8

***Scopus***

Records: 535 *(with limits of English studies)*

( ( TITLE-ABS-KEY ( sleep OR nap OR insomnia OR rest W/1 activity OR insomnia OR circadian OR "diurnal rhythm" OR "body clock" OR "biological rhythm" OR "biological clock" OR chronobiology OR chronotype OR "light exposure" OR zeitgeber OR melatonin OR "core body temperature" OR "sunlight" ) ) OR ( INDEXTERMS ( sleep ) ) ) AND ( ( TITLE-ABS-KEY ( athlete* OR sports* OR olympian OR players ) ) OR ( INDEXTERMS ( athlete ) ) ) AND ( ( INDEXTERMS ( "mental health" ) ) OR ( TITLE-ABS-KEY ( "mental health" OR depressi* OR anxi* OR wellbeing OR mood OR "mental ill-health" OR psycholog* OR stress OR "mental state" ) ) ) AND ( ( TITLE-ABS-KEY ( interven* OR program OR trial OR baseline OR workshop OR randomiz* ) ) OR ( INDEXTERMS ( intervention ) ) ) AND ( LIMIT-TO ( LANGUAGE , "English" ) )

***SPORTDiscus via EBSCOHOST***

Records: 204 (*with limits to English studies*)

(((TI "sleep" OR AB "sleep" OR SU "sleep") OR (TI "nap" OR AB "nap" OR SU "nap") OR (TI "insomnia" OR AB "insomnia" OR SU "insomnia") OR ((TI "rest" OR AB "rest" OR SU "rest") N1 (TI "activity" OR AB "activity" OR SU "activity")) OR (TI "insomnia" OR AB "insomnia" OR SU "insomnia") OR ((TI "circadian" OR AB "circadian" OR SU "circadian") OR (TI "diurnal rhythm" OR AB "diurnal rhythm" OR SU "diurnal rhythm") OR (TI "body clock" OR AB "body clock" OR SU "body clock") OR (TI "biological rhythm" OR AB "biological rhythm" OR SU "biological rhythm") OR (TI "biological clock" OR AB "biological clock" OR SU "biological clock") OR (TI "chronobiology" OR AB "chronobiology" OR SU "chronobiology") OR (TI "chronotype" OR AB "chronotype" OR SU "chronotype") OR (TI "light exposure" OR AB "light exposure" OR SU "light exposure") OR (TI "zeitgeber" OR AB "zeitgeber" OR SU "zeitgeber") OR (TI "melatonin" OR AB "melatonin" OR SU "melatonin") OR (TI "core body temperature" OR AB "core body temperature" OR SU "core body temperature") OR (TI "sunlight" OR AB "sunlight" OR SU "sunlight"))) AND ((TI "mental health" OR AB "mental health" OR SU "mental health") OR (TI "depressi*" OR AB "depressi*" OR SU "depressi*") OR (TI "anxi*" OR AB "anxi*" OR SU "anxi*") OR (TI "wellbeing" OR AB "wellbeing" OR SU "wellbeing") OR (TI "mood" OR AB "mood" OR SU "mood") OR (TI "mental ill-health" OR AB "mental ill-health" OR SU "mental ill-health") OR (TI "psycholog*" OR AB "psycholog*" OR SU "psycholog*") OR (TI "stress" OR AB "stress" OR SU "stress") OR (TI "mental state" OR AB "mental state" OR SU "mental state")) AND ((TI "athlete" OR AB "athlete" OR SU "athlete") OR (TI "sports*" OR AB "sports*" OR SU "sports*") OR (TI "Olympian" OR AB "Olympian" OR SU "Olympian") OR (TI "players" OR AB "players" OR SU "players")) AND ((TI "interven*" OR AB "interven*" OR SU "interven*") OR (TI "program" OR AB "program" OR SU "program") OR (TI "trial" OR AB "trial" OR SU "trial") OR (TI "baseline" OR AB "baseline" OR SU "baseline") OR (TI "workshop" OR AB "workshop" OR SU "workshop") OR (TI "randomiz*" OR AB "randomiz*" OR SU "randomiz*")))

***Ovid MEDLINE(R) and Epub Ahead of Print, In-Process, In-Data-Review & Other Non-Indexed Citations, Daily and Versions***

Records: 380

| # | Query | Results from 23 Sep 2024 |
| --- | --- | --- |
| 1 | sleep/ or sleep duration/ or sleep hygiene/ or sleep latency/ | 73,011 |
| 2 | sleep.ti,ab,kf. | 233,605 |
| 3 | nap.ti,ab,kf. | 6,370 |
| 4 | insomnia.ti,ab,kf. | 32,959 |
| 5 | (rest adj1 activity).ti,ab,kf. | 1,664 |
| 6 | circadian rhythm/ or chronotype/ | 78,953 |
| 7 | circadian.ti,ab,kf. | 64,019 |
| 8 | "diurnal rhythm".ti,ab,kf. | 2,878 |
| 9 | "body clock".ti,ab,kf. | 135 |
| 10 | "biological rhythm".ti,ab,kf. | 531 |
| 11 | "biological clock".ti,ab,kf. | 1,865 |
| 12 | chronobiology.ti,ab,kf. | 1,948 |
| 13 | chronotype.ti,ab,kf. | 2,579 |
| 14 | "light exposure".ti,ab,kf. | 9,194 |
| 15 | zeitgeber.ti,ab,kf. | 1,420 |
| 16 | melatonin.ti,ab,kf. | 31,765 |
| 17 | "core body temperature".ti,ab,kf. | 3,531 |
| 18 | "sunlight".ti,ab,kf. | 20,992 |
| 19 | 1 or 2 or 3 or 4 or 5 or 6 or 7 or 8 or 9 or 10 or 11 or 12 or 13 or 14 or 15 or 16 or 17 or 18 | 398,764 |
| 20 | Mental Health/ | 69,128 |
| 21 | "mental health".ti,ab,kf. | 258,114 |
| 22 | mood.ti,ab,kf. | 98,559 |
| 23 | depressi*.ti,ab,kf. | 530,141 |
| 24 | anxi*.ti,ab,kf. | 314,172 |
| 25 | Psychological Well-Being/ | 539 |
| 26 | wellbeing.ti,ab,kf. | 35,383 |
| 27 | "mental ill-health".ti,ab,kf. | 1,532 |
| 28 | psycholog*.ti,ab,kf. | 402,601 |
| 29 | stress.ti,ab,kf. | 1,070,361 |
| 30 | "mental state".ti,ab,kf. | 27,168 |
| 31 | 20 or 21 or 22 or 23 or 24 or 25 or 26 or 27 or 28 or 29 or 30 | 2,153,829 |
| 32 | Athletes/ | 22,664 |
| 33 | athlete*.ti,ab,kf. | 74,235 |
| 34 | sports*.ti,ab,kf. | 79,455 |
| 35 | olympian.ti,ab,kf. | 68 |
| 36 | players.ti,ab,kf. | 61,561 |
| 37 | 32 or 33 or 34 or 35 or 36 | 178,117 |
| 38 | interven*.ti,ab,kf. | 1,532,422 |
| 39 | program.ti,ab,kf. | 577,749 |
| 40 | trial.ti,ab,kf. | 840,895 |
| 41 | baseline.ti,ab,kf. | 785,444 |
| 42 | workshop.ti,ab,kf. | 37,605 |
| 43 | randomi*.ti,ab,kf. | 887,490 |
| 44 | 38 or 39 or 40 or 41 or 42 or 43 | 3,494,397 |
| 45 | 19 and 31 and 37 and 44 | 380 |

***EMBASE***

Records: 539

| # | Query | Results from 23 Sep 2024 |
| --- | --- | --- |
| 1 | sleep/ or sleep duration/ or sleep hygiene/ or sleep latency/ | 169,423 |
| 2 | sleep.ti,ab,kf. | 363,407 |
| 3 | nap.ti,ab,kf. | 8,899 |
| 4 | insomnia.ti,ab,kf. | 56,646 |
| 5 | (rest adj1 activity).ti,ab,kf. | 2,396 |
| 6 | circadian rhythm/ or chronotype/ | 111,416 |
| 7 | circadian.ti,ab,kf. | 84,965 |
| 8 | "diurnal rhythm".ti,ab,kf. | 3,963 |
| 9 | "body clock".ti,ab,kf. | 195 |
| 10 | "biological rhythm".ti,ab,kf. | 949 |
| 11 | "biological clock".ti,ab,kf. | 2,574 |
| 12 | chronobiology.ti,ab,kf. | 2,958 |
| 13 | chronotype.ti,ab,kf. | 4,011 |
| 14 | "light exposure".ti,ab,kf. | 11,348 |
| 15 | zeitgeber.ti,ab,kf. | 1,906 |
| 16 | melatonin.ti,ab,kf. | 40,140 |
| 17 | "core body temperature".ti,ab,kf. | 4,725 |
| 18 | "sunlight".ti,ab,kf. | 24,599 |
| 19 | 1 or 2 or 3 or 4 or 5 or 6 or 7 or 8 or 9 or 10 or 11 or 12 or 13 or 14 or 15 or 16 or 17 or 18 | 586,505 |
| 20 | Mental Health/ | 242,447 |
| 21 | "mental health".ti,ab,kf. | 317,396 |
| 22 | mood.ti,ab,kf. | 147,919 |
| 23 | depressi*.ti,ab,kf. | 750,057 |
| 24 | anxi*.ti,ab,kf. | 452,089 |
| 25 | Psychological Well-Being/ | 35,661 |
| 26 | wellbeing.ti,ab,kf. | 50,076 |
| 27 | "mental ill-health".ti,ab,kf. | 1,869 |
| 28 | psycholog*.ti,ab,kf. | 543,355 |
| 29 | stress.ti,ab,kf. | 1,358,362 |
| 30 | "mental state".ti,ab,kf. | 39,544 |
| 31 | 20 or 21 or 22 or 23 or 24 or 25 or 26 or 27 or 28 or 29 or 30 | 2,891,614 |
| 32 | Athletes/ | 65,698 |
| 33 | athlete*.ti,ab,kf. | 90,073 |
| 34 | sports*.ti,ab,kf. | 105,504 |
| 35 | olympian.ti,ab,kf. | 96 |
| 36 | players.ti,ab,kf. | 72,690 |
| 37 | 32 or 33 or 34 or 35 or 36 | 229,936 |
| 38 | interven*.ti,ab,kf. | 2,132,463 |
| 39 | program.ti,ab,kf. | 817,121 |
| 40 | trial.ti,ab,kf. | 1,249,431 |
| 41 | baseline.ti,ab,kf. | 1,351,425 |
| 42 | workshop.ti,ab,kf. | 52,723 |
| 43 | randomi*.ti,ab,kf. | 1,277,213 |
| 44 | 38 or 39 or 40 or 41 or 42 or 43 | 5,142,945 |
| 45 | 19 and 31 and 37 and 44 | 539 |

***PsycINFO***

Records: 71 *(with limits of human and English studies)*

| # | Query | Results from 23 Sep 2024 |
| --- | --- | --- |
| 1 | sleep/ or sleep duration/ or sleep hygiene/ or sleep latency/ | 31,274 |
| 2 | sleep.ti,ab,id. | 92,689 |
| 3 | nap.ti,ab,id. | 1,440 |
| 4 | insomnia.ti,ab,id. | 16,775 |
| 5 | (rest adj1 activity).ti,ab,id. | 771 |
| 6 | circadian rhythm/ or chronotype/ | 1,075 |
| 7 | circadian.ti,ab,id. | 16,113 |
| 8 | "diurnal rhythm".ti,ab,id. | 530 |
| 9 | body clock.ti,ab,id. | 49 |
| 10 | "biological rhythm".ti,ab,id. | 219 |
| 11 | "biological clock".ti,ab,id. | 454 |
| 12 | chronobiology.ti,ab,id. | 679 |
| 13 | chronotype.ti,ab,id. | 1,632 |
| 14 | "light exposure".ti,ab,id. | 1,276 |
| 15 | zeitgeber.ti,ab,id. | 477 |
| 16 | melatonin.ti,ab,id. | 5,477 |
| 17 | "core body temperature".ti,ab,id. | 617 |
| 18 | "sunlight".ti,ab,id. | 778 |
| 19 | 1 or 2 or 3 or 4 or 5 or 6 or 7 or 8 or 9 or 10 or 11 or 12 or 13 or 14 or 15 or 16 or 17 or 18 | 114,536 |
| 20 | Athletes/ | 17,097 |
| 21 | athlete*.ti,ab,id. | 21,846 |
| 22 | olympian.ti,ab,id. | 60 |
| 23 | sports*.ti,ab,id. | 29,010 |
| 24 | players.ti,ab,id. | 20,899 |
| 25 | 20 or 21 or 22 or 23 or 24 | 59,018 |
| 26 | Mental Health/ | 100,084 |
| 27 | "mental health".ti,ab,id. | 263,566 |
| 28 | mood.ti,ab,id. | 87,118 |
| 29 | depress*.ti,ab,id. | 380,115 |
| 30 | anxi*.ti,ab,id. | 270,002 |
| 31 | well being/ or subjective well being/ | 67,495 |
| 32 | wellbeing.ti,ab,id. | 25,579 |
| 33 | "mental ill-health".ti,ab,id. | 1,390 |
| 34 | psycholog*.ti,ab,id. | 698,594 |
| 35 | stress.ti,ab,id. | 274,850 |
| 36 | "mental state".ti,ab,id. | 17,476 |
| 37 | 26 or 27 or 28 or 29 or 30 or 31 or 32 or 33 or 34 or 35 or 36 | 1,515,501 |
| 38 | Intervention/ | 94,406 |
| 39 | intervention.ti,ab,id. | 324,230 |
| 40 | program.ti,ab,id. | 266,652 |
| 41 | trial.ti,ab,id. | 136,144 |
| 42 | baseline.ti,ab,id. | 150,200 |
| 43 | workshop.ti,ab,id. | 12,989 |
| 44 | randomi*.ti,ab,id. | 118,663 |
| 45 | 38 or 39 or 40 or 41 or 42 or 43 or 44 | 785,536 |
| 46 | 19 and 25 and 37 and 45 | 79 |
| 47 | limit 46 to (human and english language) | 71 |

***CINAHL via EBSCOHOST***

Records: 255

| S1 | sleep | Search modes - Proximity | 91,843 |
| --- | --- | --- | --- |
| S2 | (((TI sleep OR AB sleep OR SU sleep) OR (TI nap OR AB nap OR SU nap) OR (TI insomnia OR AB insomnia OR SU insomnia) OR ((TI rest OR AB rest OR SU rest) N1 (TI activity OR AB activity OR SU activity)) OR (TI insomnia OR AB insomnia OR SU insomnia) OR ((TI circadian OR AB circadian OR SU circadian) OR (TI "diurnal rhythm" OR AB "diurnal rhythm" OR SU "diurnal rhythm") OR (TI "body clock" OR AB "body clock" OR SU "body clock") OR (TI "biological rhythm" OR AB "biological rhythm" OR SU "biological rhythm") OR (TI "biological clock" OR AB "biological clock" OR SU "biological clock") OR (TI chronobiology OR AB chronobiology OR SU chronobiology) OR (TI chronotype OR AB chronotype OR SU chronotype) OR (TI "light exposure" OR AB "light exposure" OR SU "light exposure") OR (TI zeitgeber OR AB zeitgeber OR SU zeitgeber) OR (TI melatonin OR AB melatonin OR SU melatonin) OR (TI "core body temperature" OR AB "core body temperature" OR SU "core body temperature") OR (TI sunlight OR AB sunlight OR SU sunlight))) | Search modes - Proximity | 115,182 |
| S3 | S1 OR S2 | Search modes - Proximity | 115,182 |
| S4 | mental health | Search modes - Proximity | 202,392 |
| S5 | ((TI "mental health" OR AB "mental health" OR SU "mental health") OR (TI depressi* OR AB depressi* OR SU depressi*) OR (TI anxi* OR AB anxi* OR SU anxi*) OR (TI wellbeing OR AB wellbeing OR SU wellbeing) OR (TI mood OR AB mood OR SU mood) OR (TI "mental ill-health" OR AB "mental ill-health" OR SU "mental ill-health") OR (TI psycholog* OR AB psycholog* OR SU psycholog*) OR (TI stress OR AB stress OR SU stress) OR (TI "mental state" OR AB "mental state" OR SU "mental state")) | Search modes - Proximity | 879,866 |
| S6 | S4 OR S5 | Search modes - Proximity | 882,801 |
| S7 | athlete | Search modes - Proximity | 55,738 |
| S8 | MM athlete | Search modes - Proximity | 0 |
| S9 | ((TI athlete OR AB athlete OR SU athlete) OR (TI sports* OR AB sports* OR SU sports*) OR (TI Olympian OR AB Olympian OR SU Olympian) OR (TI players OR AB players OR SU players)) | Search modes - Proximity | 114,064 |
| S10 | intervention | Search modes - Proximity | 588,902 |
| S11 | ((TI interven* OR AB interven* OR SU interven*) OR (TI program OR AB program OR SU program) OR (TI trial OR AB trial OR SU trial) OR (TI baseline OR AB baseline OR SU baseline) OR (TI workshop OR AB workshop OR SU workshop) OR (TI randomiz* OR AB randomiz* OR SU randomiz*)) | Search modes - Proximity | 1,674,900 |
| S12 | S10 OR S11 | Search modes - Proximity | 1,674,900 |
| S13 | S7 OR S9 | Search modes - Proximity | 114,064 |
| S14 | S3 AND S6 AND S12 AND S13 | Search modes - Proximity | 255 |

**Table S3, Quality Assessment Tools**

| **Table S3a, Quality assessment using ROB** | | | |
| --- | --- | --- | --- |
|  | **Ritland et al. 2019** | **Fowler et al. 2021** | **Harris et al. 2015** |
| Bias arising from the randomisation process | Some concerns | Some concerns | Some concerns |
| Bias due to deviations from intended interventions | Some concerns | Some concerns | High |
| Bias due to missing outcome data | High | High | Low |
| Bias in measurement of the outcome | Some concerns | Some concerns | Some concerns |
| Bias in selection of the reported results | Some concerns | Some concerns | Some concerns |
| **Overall  bias** | Some concerns | Some concerns | Some concerns |

| **Table S3b, Quality assessment using ROB cross-over** | | | | | |
| --- | --- | --- | --- | --- | --- |
|  | **Bentouati et al. 2023** | **Fullagar et al. 2016** | **Lever et al. 2020** | **Souabni et al. 2022** | **Roberts et al. 2018** |
| Randomisation process | Some concerns | Some concerns | Some concerns | Low | Some concerns |
| Risk of bias arising from period and carryover effects | Some concerns | Some concerns | High | Some concerns | High |
| Deviations from intended interventions | High | High | High | Some concerns | High |
| Missing outcome data | High | High | Low | High | High |
| Measurement of the outcome | Some concerns | High | High | Some concerns | Some concerns |
| Selection of the reported result | High | High | High | Some concerns | Some concerns |
| **Overall Bias** | ***High*** | ***High*** | ***High*** | ***Some concerns*** | ***High*** |

| **Table S3c, Quality assessment using JBI** | | | | | |
| --- | --- | --- | --- | --- | --- |
|  | **Van Ryswyk et al. 2017** | **Mah et al. 2011** | **Harada et al. 2016** | **Bonnar et al. 2022** | **Boukhris et al. 2020** |
| **Q1** | Y | Y | Y | Y | Y |
| **Q2** | Y | Y | Y | Y | Y |
| **Q3** | Y | Y | Y | Y | Y |
| **Q4** | N | N | N | N | Y |
| **Q5** | N | N | N | N | N |
| **Q6** | Y | Y | NA | NA | Y |
| **Q7** | Y | Y | Y | Y | Y |
| **Q8** | Y | Y | N | Y | Y |
| **Q9** | Y | Y | N | Y | Y |
| **Overall** | *Include* | *Include* | *Include* | *Include* | *Include* |
| **Total Y** | 77.78% | 77.78% | 44.44% | 66.67% | 88.89% |

**Table S4, Instruments used for the measurement of mental health outcomes in selected studies**

| **Instruments** | **No. (Percentage of articles %*)** | **Outcome** | **Description** |
| --- | --- | --- | --- |
| POMS- Profile of Mood States | 11(52.4%) | Mood | The Profile of Mood States (POMS) is a self-report measure assessing short-term, fluctuating mood states. The measure includes six subscales and yields a Total Mood Disturbance (TMD) score by summing negative subscales and subtracting vigour, providing an overall index of mood disturbance. |
| BRUMS- Brunel Mood Scale | 1(4.8%) | Mood; Vigour | The Brunel Mood Scale (BRUMS) is a psychological assessment tool designed to measure mood states. The BRUMS assesses six mood dimensions: Tension, depression, anger, vigour, fatigue, confusion. Higher scores indicate stronger feelings or higher intensity of the mood states measured. |
| Centre for Epidemiological Studies Depression (CES-D) | 1(4.8%) | Depression | The Centre for Epidemiological Studies Depression Scale (CES-D) is a widely used self-report depression scale designed to measure depressive symptomatology in the general population. The total score ranges from 0 to 60, with higher scores indicating more severe depressive symptoms. |
| Competitive State Anxiety (CSAI-2) | 1(4.8%) | Anxiety | The Competitive State Anxiety Inventory (CSAI) is a tool designed to measure the anxiety levels of athletes in competitive settings. It specifically assesses three components of anxiety: cognitive, somatic and self-confidence. There are 27 items with higher scores indicating greater levels of anxiety or confidence. |
| General Anxiety Disorder (GAD-7) | 1(4.8%) | Anxiety | The Generalized Anxiety Disorder 7 (GAD-7) is a brief self-report questionnaire used to screen for and measure the severity of a generalized anxiety disorder. Higher scores indicate more severe anxiety. |
| State-Trait Anxiety Inventory (STAI-Y) | 1(4.8%) | Anxiety | The State-Trait Anxiety Inventory (STAI) measures state and trait anxiety in adults. Higher scores indicate higher levels of anxiety. |
| Motivation Likert scale | 2(9.5%) | Motivation | A Motivation Likert scale is a tool used to measure an individual's level of motivation. Higher scores indicate higher motivation. |
| Perceptual Fatigue and Recovery questionnaire | 1(4.8%) | Stress | The Perceptual Fatigue and Recovery Questionnaire is a tool designed to assess an individual's subjective experience of fatigue and recovery. Higher scores indicate higher levels of fatigue or better recovery. |
| Irritation Index | 1(4.8%) | Irritation | The Irritation Index is a psychological assessment tool used to measure an individual's level of irritation or annoyance. Higher scores indicate higher levels of irritation. |
| General Health Questionnaire (GHQ) | 1(4.8%) | Mental health | The General Health Questionnaire (GHQ) is a widely used screening tool designed to detect psychiatric disorders and assess general mental health. Higher scores indicate greater psychological distress. |
| Perceptual Wellbeing questionnaire | 1(4.8%) | Wellbeing | The Perceptual Wellbeing Questionnaire is a tool designed to assess an individual’s subjective experience of wellbeing, focusing on various dimensions of psychological and emotional health. Higher scores generally indicate a higher level of perceived wellbeing. |
| Wellness and Mood states | 1(4.8%) | Wellbeing; Mood | NA |
| Positive and Negative Affect Schedule (PANAS) | 1(4.8%) | Affect | The Positive and Negative Affect Schedule (PANAS) is a widely used psychological assessment tool designed to measure positive and negative affectivity. Higher scores indicate higher levels of positive emotions on the positive affect score. On the negative affect score, higher scores indicate higher levels of negative emotions. |

**S5 Meta analysis: Forrest Plots**

Figure S5.1 Forrest Plot illustrating the effect of sleep and circadian interventions on “Mood”
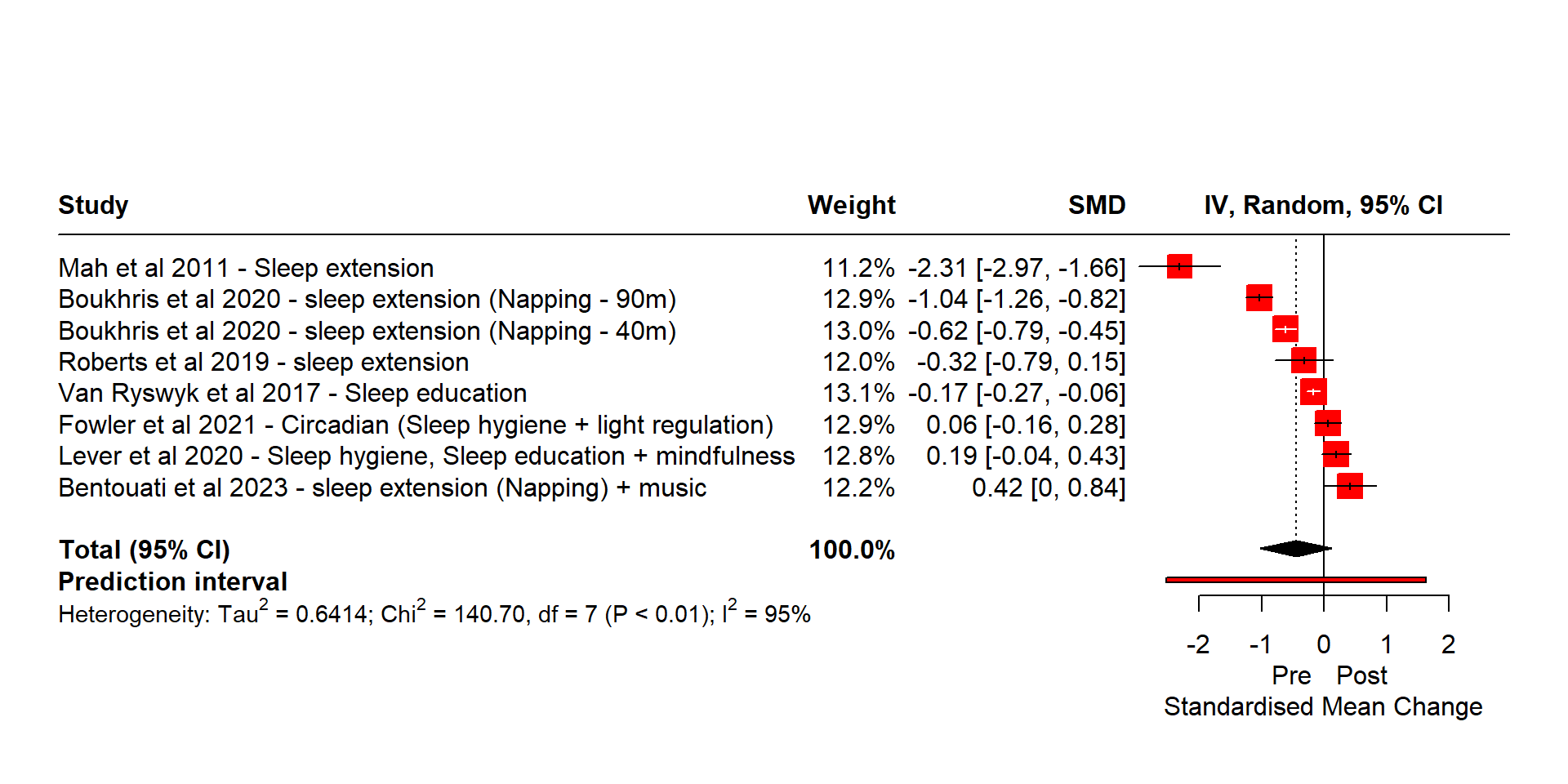


Figure S5.2 Forrest Plot illustrating the effect of sleep interventions on “Tension”


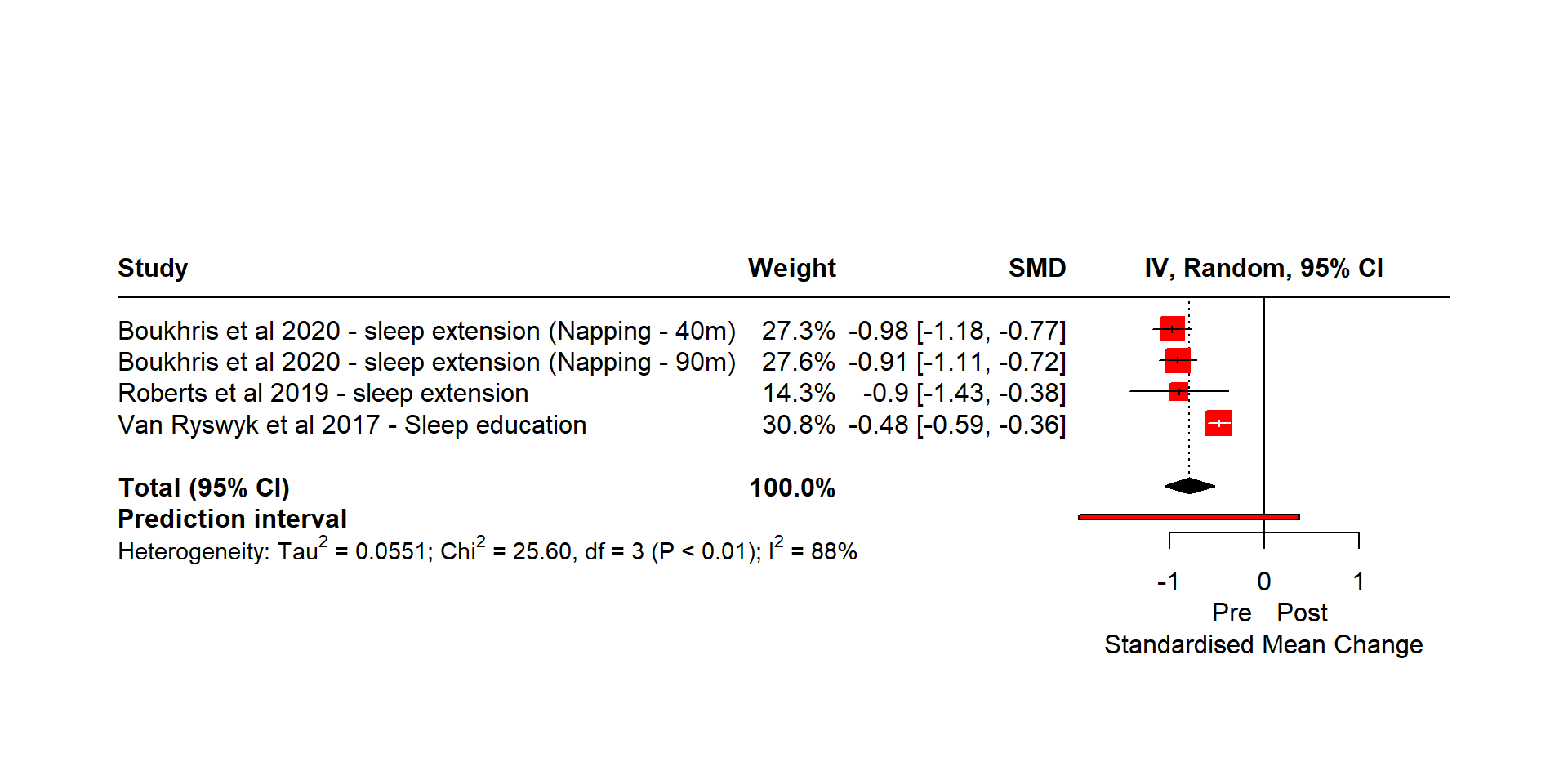


Figure S5.3 Forrest Plot illustrating the effect of sleep interventions on “Vigour”


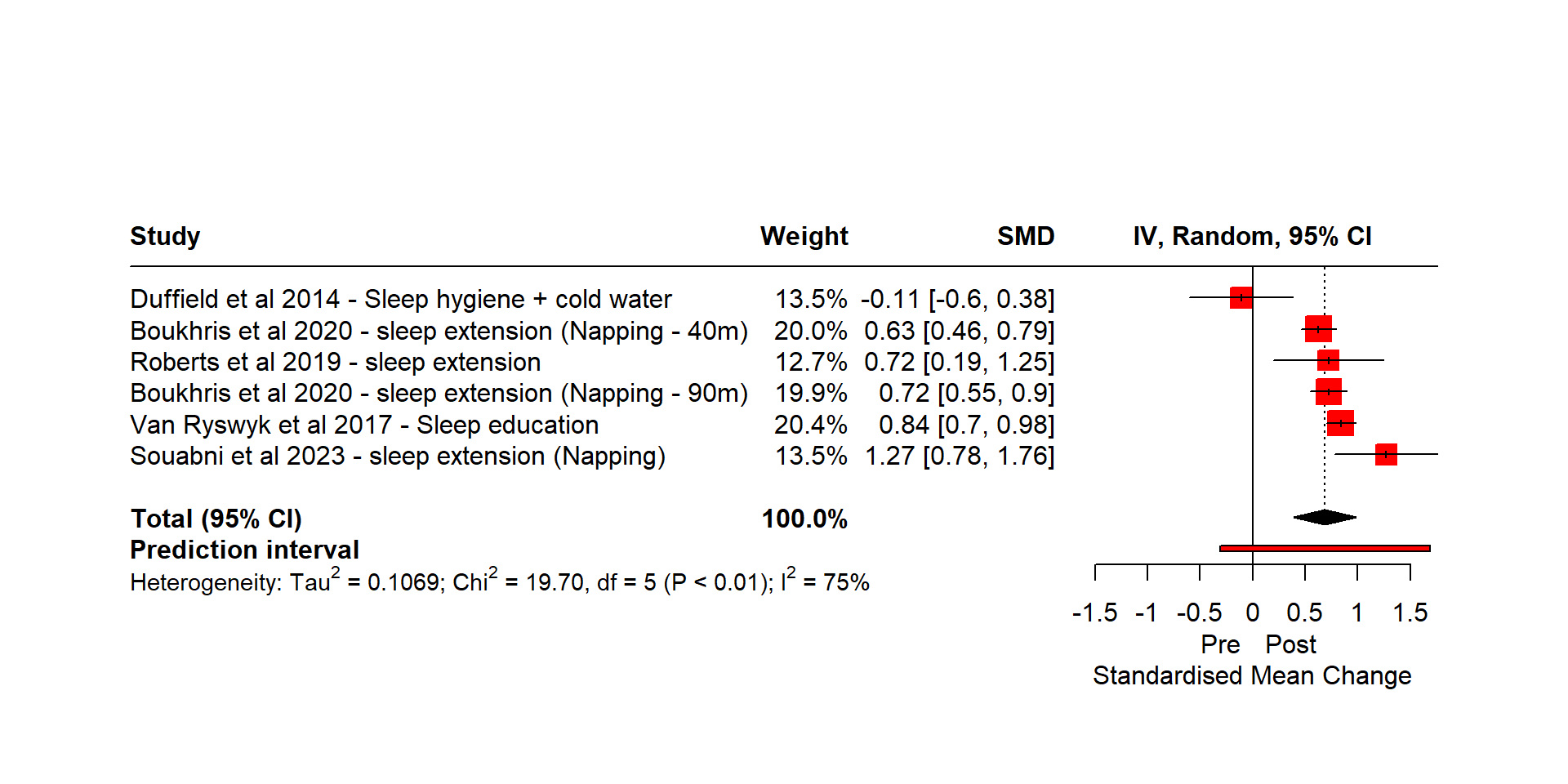


Figure S5.4 Forrest Plot illustrating the effect of sleep interventions on “Confusion”
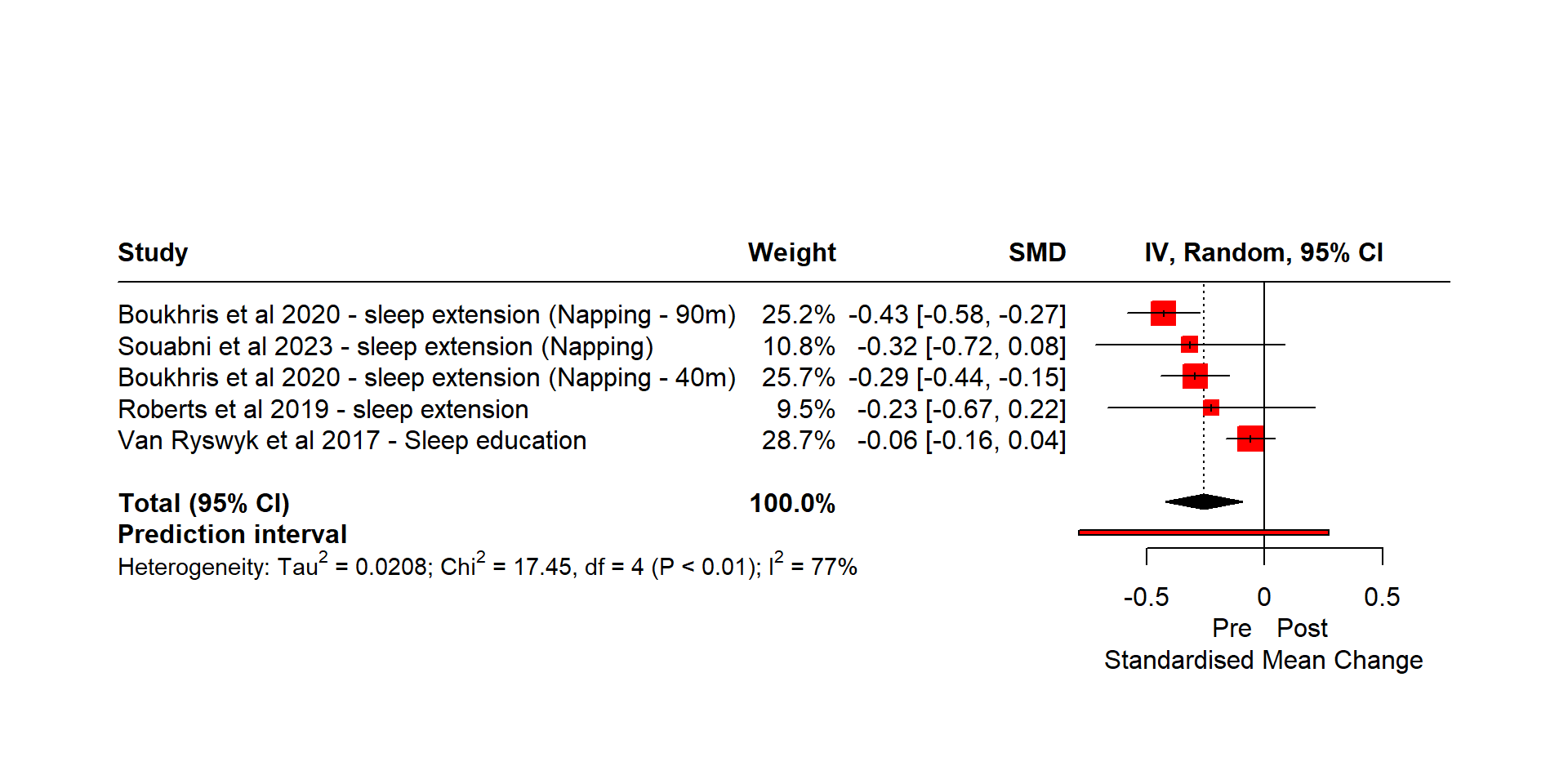


Figure S5.5 Forrest Plot illustrating the effect of sleep and circadian interventions on “Anxiety”
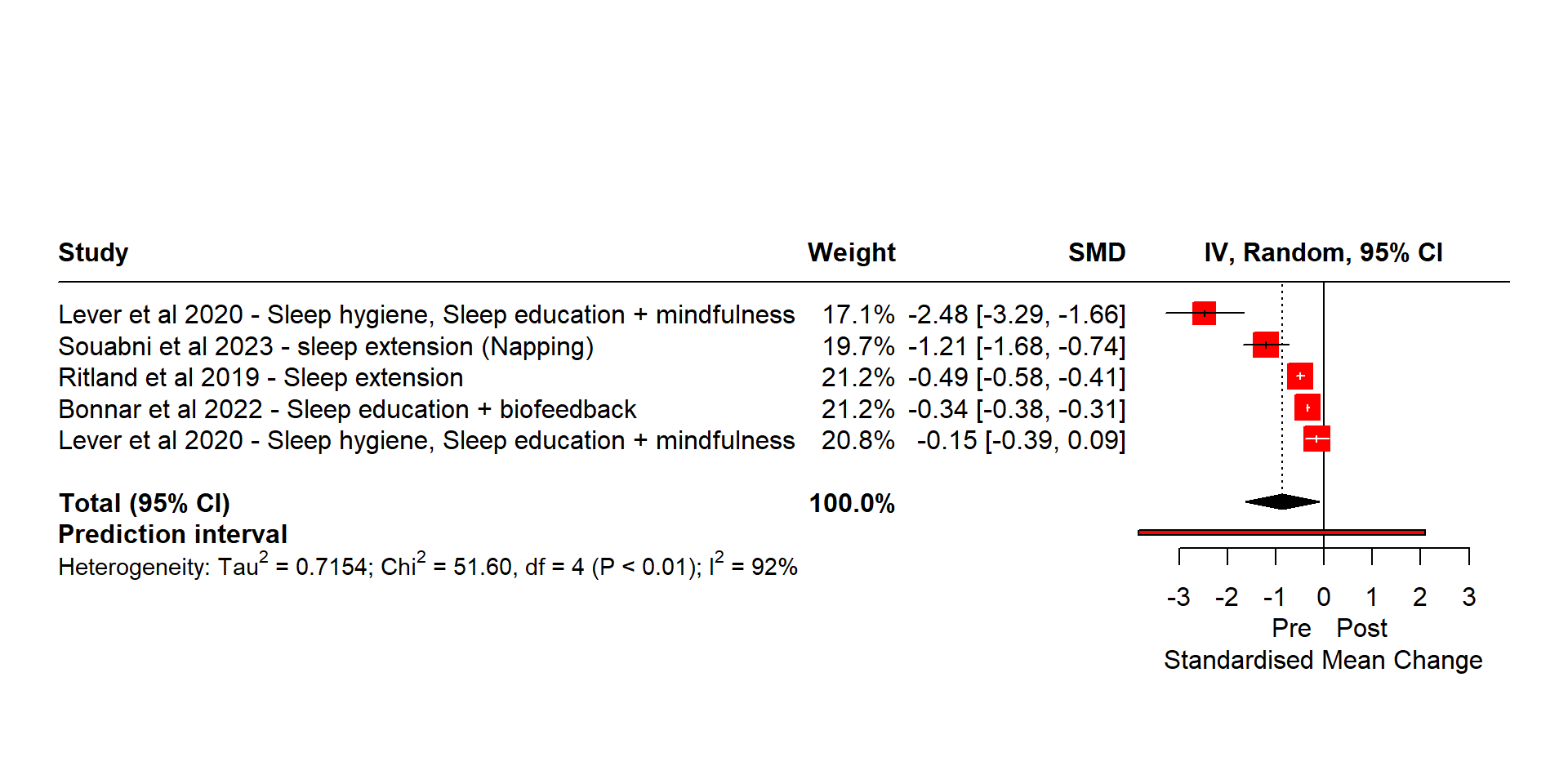


Figure S5.6 Forrest Plot illustrating the effect of sleep interventions on “Anger”


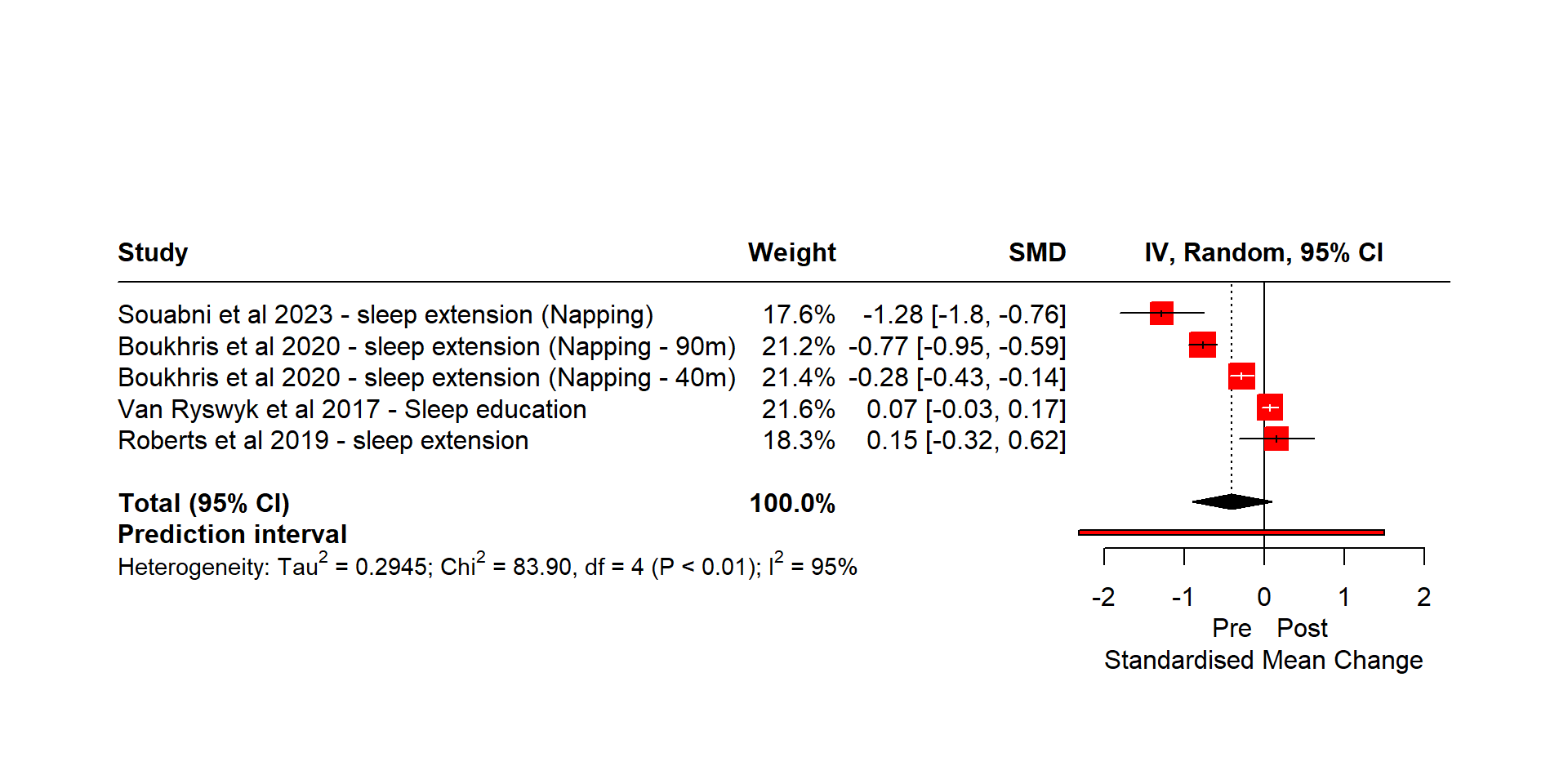


Figure S5.7 Forrest Plot illustrating the effect of sleep and circadian interventions on “Worry and Stress”


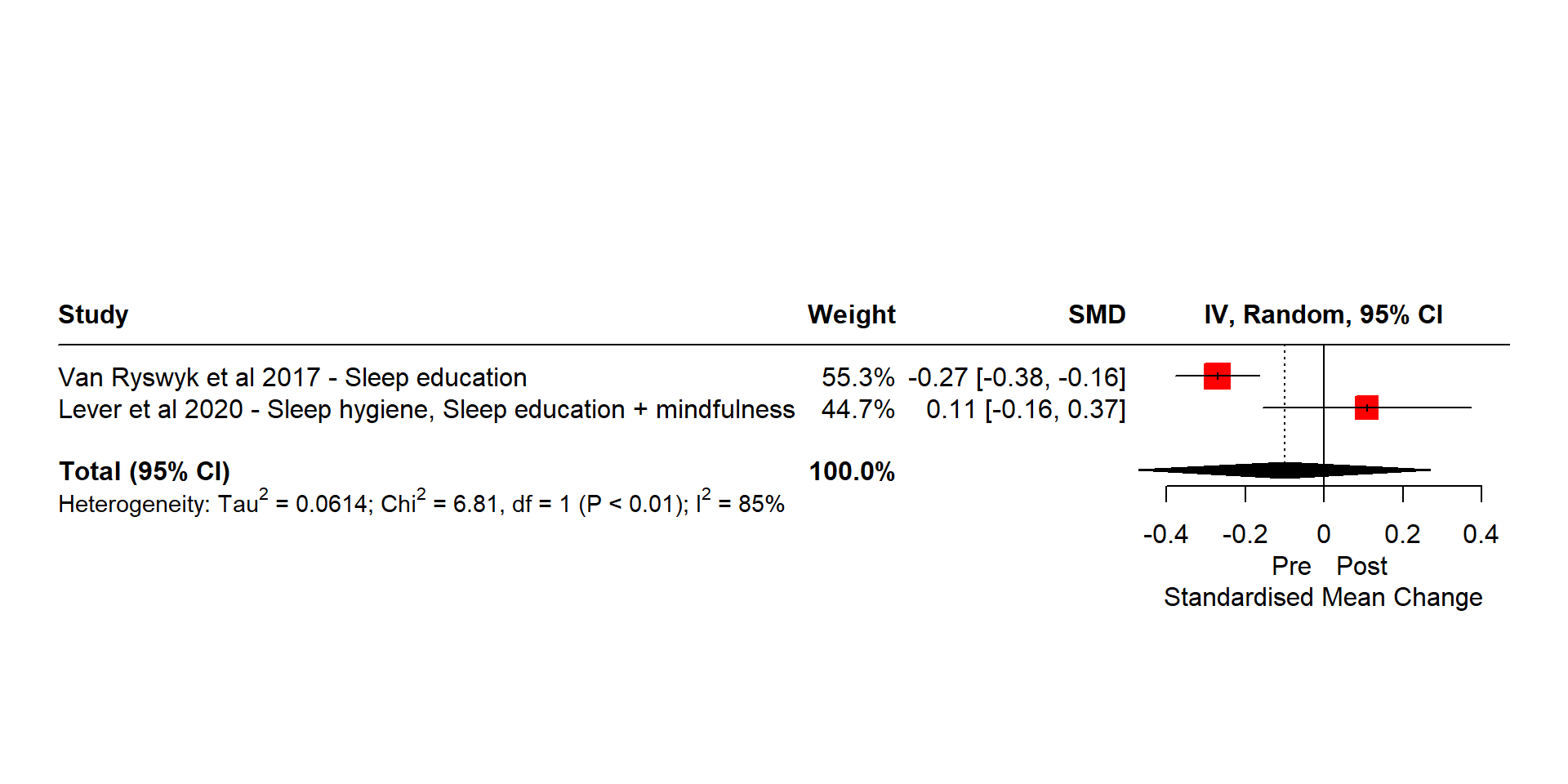


Figure S5.8 Forrest Plot illustrating the effect of sleep interventions on “Depression”


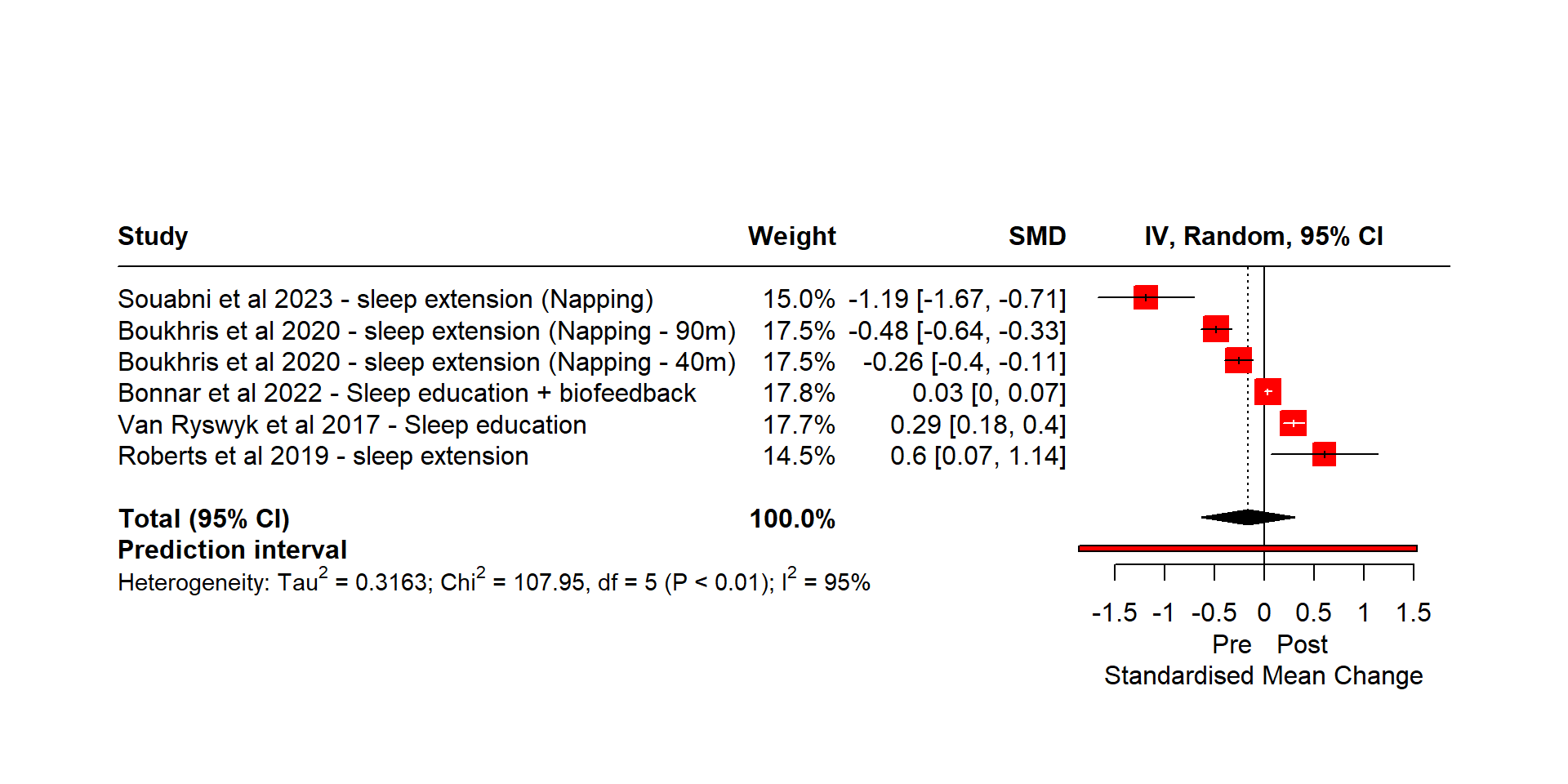

Supplement: Supplementary file 2 — Supplementary file2 (DOCX 3475 KB) [file 40279_2025_2387_MOESM2_ESM.docx]
