# Supplementary material for: Sleep and Circadian Interventions to Improve Athletes’ Mental Health, Mood and Well-Being: A Systematic Review and Meta-Analysis
Source: Sports Med. 2026 Jan 27;56(4):997–1011. doi: 10.1007/s40279-025-02387-z (PMC13124852; doi:10.1007/s40279-025-02387-z)
Supplement: Supplementary file 3 — Supplementary file3 (DOCX 54 KB) [file 40279_2025_2387_MOESM3_ESM.docx]

**Table S2, Data Extraction Table**

| **Author/Date** | **Study design; Article type** | **Intervention type** | **Intervention components** | **Intervention characteristics** | **Intervention length/ follow up** | **Location** | **Sport type** | **Participants, (% female), Group sample age** | **Outcomes** | **Measurements** | **Key results** | **Successfully manipulated sleep** |
| --- | --- | --- | --- | --- | --- | --- | --- | --- | --- | --- | --- | --- |
| Bender et al 2017 | single group pre-post; *conference abstract* | Sleep and Circadian | sleep extension; electronic device restriction; blue light blocking glasses | **Group 1 (curlers)** *:* INT: Combined sleep extension, napping, and restricting electronics 1hr before bed **Group 2 (rowers):** INT + blue-blocking glasses (2hrs before bed) | 3.5 wks (24.5 days) | Canada | curling; rowing | **Group 1**: 15(53%%), 30.7 y ± 4.5 **Group 2**: 11 (100%), 26.0 y ± 3.1 | mood | POMS | Group 1: Reduced fatigue, total mood disturbance, and increased vigour following intervention (*p*<.05) Group 2**:** Reduced depressive mood and total mood disturbance following intervention (*p*<.05) *Differences between groups not listed* | NA |
| Athley et al 2017 | single group pre-post; *conference abstract* | Sleep | sleep education | 90min sleep education session, 24/7 access to peer support and study staff 10 wk follow up | 10 wks (70 days) | US | NA | *n* = 35 | stress, social life, family life, mental health, energy level (*qualitative*), anxiety | Survey; Generalised Anxiety Disorder (GAD-7) Scale | Positive changes in mental health, stress, social life, family life and energy levels were reported in 66-91% of the sample. Lower GAD score *(p* = 0.025) was found pre-post intervention | NA |
| Boland et al 2023 | randomised crossover study; *conference abstract* | Sleep | sleep consistency; sleep extension | 2-week sleep consistency intervention *2-week washout* 2-week sleep extension intervention | 2 wks (14 days) | US | soccer; water polo | **overall** (*n)*= 46 **soccer**: 21 (62%) 18-22 y **water polo**: 25(0%) 18-22 y | mood | POMS | There was no change in mood state with any intervention | NA |
| Charest et al 2017 | single group pre-post; *conference abstract* | Sleep and Circadian | sleep extension; electronic device restriction; blue light blocking glasses | sleep optimization intervention (2 weeks) which included daily napping, increasing nighttime sleep, and a bedtime routine which included an electronic device curfew and wearing blue light blocking glasses 2 hrs before bedtime | 2 wks (14 days) | Canada | speed skaters | n = 7 (43%), 24.3 y ± 4.2 | mood | POMS | Increased vigour (*p*<.05) and reduced total mood symptoms (*p* =.05) | Y |
| Duffield et al 2014 | cross over study | Sleep | cold water; compression garments; sleep hygiene | **INT:** Combined 15min cold water immersion, 3h of full-body compression garment and sleep hygiene recommendations (low light environment by 21:00, limit devices, light luminescence of 3-8lux) **CG:** post-session stretching and no sleep regulations ***One night*** | 1 day | US | tennis | n = 8 (0%), 20.9 y ± 3.6 | vigour | BRUMS | No differences in vigour | N |
| Famodu et al 2017 | single group pre-post; *conference abstract* | Sleep | sleep extension only | 1 week baseline 1 week of sleep ext (up to 60 minutes) | 1 wk (7 days) | US | track | n = 15(100%), 20 y ± 2 | mood | POMS | POMS total mood disturbance scores decreased (*p*<.05) | Y |
| Mah et al 2017 | randomised control trial; *conference abstract* | Sleep | sleep extension only | 2 day baseline **INT**: sleep extension up to 10 hrs for 5 nights.  **CG**: maintain habitual sleep | 5 days | US | baseball | n = 17(0%) | tension | POMS | Tension significantly decreased in the intervention group (*p* = .04). No other information for the other subscale scores. | Y |
| Skein et al 2017 | randomised cross over; *conference abstract* | Sleep | sleep extension only | Group 1 (**CG**): normal night sleep (7-8 hours) Group 2: shortened sleep duration (4 hours) Group 3 **INT**: extended sleep (10 hours) Group 4 : sleep fragmentation (FRAG) including 7-8 hours sleep but with alarms set every 3 hours during the night ***One night*** | 1 day | NA | NA | n = 15, 14-17 y | wellbeing; mood | Wellness and Mood States questionnaires | Wellness and mood states were negatively affected in restriction and fragmented sleep compared to CG. ***No available information on the effect of Sleep Ext intervention.*** | NA |
| Bentouati et al 2023 | randomised, counter balanced cross over | Sleep | napping; music | Group 1 **(CG):** 30-min nap opportunity (N30) Group 2: a warm-up with self-selected motivational music (WUMM) Group 3: WUMM + N30 *1 week washout* ***One day*** | 1 day | NA | karate | n = 14(0%), 19.85 y ± 2.07 | mood | POMS | All interventions improved their mood (*p*<.05). No differences between warm up and nap conditions. | Y |
| Bonnar et al 2022 | single group pre-post | Sleep | sleep education; biofeedback | Group sleep education class, 1:1 session with a trained clinical psychologist and daily biofeedback | 2 wks (14 days) | South Korea, US, Australia | esports | n = 56 (4%), 20.9 y ± 2.43 | depression; anxiety | Centre for Epidemiological Studies Depression (CES-D); State-Trait Anxiety Inventory (STAI-Y) | No significant change in mood. Anxiety (*F*= 1.03, *p* = 0.31). Depression (*F*= 0.01, *p* = 0.94). | Y |
| Fowler et al 2021 | randomised matched pairs | Sleep and Circadian | sleep; circadian; light; jet lag; blue bight blocking glasses | **INT:** Sleep hygiene during flight (maximize sleep from certain times, sleep within biological night time, restrict devices, assisted sleep tools such as mask, pillow); light controlled for on arrival **CG**: Normal routine | 4 days | Australia | NA | n = 20(0%), 21.9 y ± 3.6 | mood; motivation | BRUMS; Motivation Likert scale | Mood and motivation were worse in the control group. The intervention group reported no change in mood pre/post flight. | Y |
| Fullagar et al 2016 | randomised cross-over design | Sleep | sleep hygiene; recommendations to restrict light | **INT**: Acute sleep hygiene strategy after a late night match and restriction of light before bed (no technological or light stimulation was allowed ~15–30 min prior to bedtime, optimize sleep environment, sleep window) **CG:** normal post-game routine | 1 day | Germany | soccer | n = 20(0%) | stress | Perceptual fatigue and recovery questionnaire | No differences in overall stress (*p* = 0.94) | Y |
| Harada et al 2016 | single group pre-post | Sleep and Circadian | sleep and circadian education; light | Leaflet to promote a morning type lifestyle for one month. Recommended to follow 8 actions: early morning exposure to sunlight (1), sunlight exposure after breakfast (2), use of lighting emitting low-color-temperature lights (e.g., incandescent lamp) (3), protein rich breakfast (4), limitation of watching TV at night (5), limitation of PC use at night (6), limitation of playing electric game at night (7), recording of sleep diary (8) | 3 wks (28 days) | Japan | soccer | n = 84(0%), 18-22 y | irritation; mental health | Irritation index; General Health Questionnaire (GHQ) | Irritation reduced after the intervention (*p* = 0.006) | Y |
| Harris et al 2015 | randomised control trial | Sleep and Circadian | electronic restriction | **INT:** 4 weeks with restricted use of electronic media after 22:00 **CG:** normal behaviour | 4 wks (28 days) | NA | NA | n = 85(40%), 16.4 y ± .08 | positive/negative affect | PANAS | No difference in mood between/within groups. Positive affect (*F* = .32 , *p* = .57) Negative affect (*F* = .06, *p* = .80) | N |
| Lever et al 2020 | randomised cross over design | Sleep | sleep education; mindfulness | **INT:** 40 minute sleep education workshop, sleep hygiene recommendations and mindfulness **CG**: Normal routine *5 day follow up* | 5 days | NA | tennis | n = 17(41%), 15.4 y ± 1.1 | anxiety; wellbeing | Perceptual wellbeing questionnaire; Competitive State Anxiety Inventory-3 | Mood and anxiety showed no significant differences between conditions. Increased feelings of worry (p*<*.05) and lower levels of confidence in the control group compared to their baseline (p*<*.05) . | Y |
| Mah et al 2011 | single group pre-post | Sleep | sleep extension only | 2-4 wk baseline 5-7 wk sleep extension, min goal of 10hr bed each night. A regular sleep-wake schedule was also encouraged and day time naps. | 5 -7 wks (35-49 days) | US | basketball | n = 11(0%), 19.4 y ± 1.4 | mood | POMS | All POMS subscale scores demonstrated improvement (*p*<.01) | Y |
| Ritland et al 2019 | randomised control trial | Sleep | sleep extension; naps | **INT**: 4 sleep ext nights, goal of spending 10hrs in bed (napping allowed if this could be achieved at night) **CG**: normal routine *7 night baseline (pretest) 4 day sleep extension (posttest) 4 habitual nights (follow up)* | 4 days | US | tactical athletes | Overall n = 50, **INT**: n = 25 (52%), 20.12 y ± 2.01 **CG**: n = 25 (48%), 19.76 y ± 1.09 | motivation; anxiety | 100-mm Visual Analog Scale (VAS) (anchors: No motivation, Highest possible motivation); State-Trait Anxiety Inventory | Motivation: Difference in motivation between two groups. Intervention had higher motivation to engage in cognitive and performance tasks (*p*<.01) Anxiety: No significant differences in anxiety levels between groups | Y |
| Roberts et al 2019 | counterbalanced crossover design | Sleep | sleep extension only | Group 1 **(INT)**: 3 nights of extension (SE) habitual "time in bed" extended by 30 % Group 2 : 3 nights if sleep restriction habitual "time in bed" reduced by 30 % Group 3 **(CG):** normal sleep *4 night baseline, 3 day intervention, 7 day washout period* | 3 days | Australia | triathlete; cyclists | n = 9 (0%), 30 y ± 6 | mood | POMS | No difference in anger, total mood, confusion, depression between day 1 and day 3 of INT or between INT and CG condition. Only significant difference was vigour on day 4 between INT and CG (*p* < 0.025) | Y |
| Souabni et al 2023 | randomised crossover design | Sleep | sleep extension; naps | **INT:** 40-minute nap opportunity **CG:** no nap condition *72 hour washout period one day* | 1 day | NA | basketball | n = 10, 27.6 y ± 4.7 | mood | POMS | Scores were significantly lower after nap compared to values before nap for anxiety, anger and fatigue (*p*<.01) Anxiety values were significantly lower after INT compared to values after CG (*p*<.01, *d* = -1.26 ) and vigour was higher (*p* = 0.03, *d* = 0.8) | Y |
| Van Ryswyk et al 2017 | single group pre-post | Sleep | sleep education | 1hr education session, feedback to improve sleep quality and quantity through weekly email and SMS, 1hr mid program education session *Follow up 6 weeks* | 6 wks (42 days) | Australia | Australian football league | n = 19(0%), 23.7 y ± 2.0 | mood; stress | POMS; Perceived Stress Scale | No differences in total mood, depression, anger, confusion, tension or stress. Vigour improved after intervention (*p*<.001). | Y *(subjective only)* |
| Boukhris et al 2020 | cross over | Sleep | naps | Group 1 **(CG)**: No nap Group 2: 40-minute nap Group 3**:** 90-minute nap *72 hr washout period immediate pre post measures, one day* | 1 day | NA | soccer; rugby; handball | n = 14 (20.3 y ± 3.0) | mood | POMS | Total mood scores were better after nap conditions compared to group 1 (CG) (*p* < 0.05), with lower scores after group 3(90 min nap) compared to group 2 (40 min nap)(*p* < 0.05). | Y |

***

*****Key**

| **Abbreviation** | **Meaning** |
| --- | --- |
| CG | Control |
| INT | Intervention |
| RCT | Randomised control trial |
| Ext | Extension |
| NA | Not available |
| wks | Weeks |
| POMS | Profile of Mood States |
| BRUMS | Brunel Mood Scale |
| GHQ | General Health Questionnaire |
| CES-D | Centre for Epidemiologic Studies Depression Scale |
| STAI-Y | State-Trait Anxiety Inventory |
| PANAS | Positive and Negative Affect Schedule |
| y | Years |
